# Influence of Calcium Ions on the Membrane Binding of S100B Protein

**DOI:** 10.1021/acs.langmuir.5c03189

**Published:** 2025-08-29

**Authors:** Paul Jaouen, Élodie Boisselier

**Affiliations:** † Department of ophthalmology and Otolaryngology - Head and Neck Surgery, Faculty of medicine, 4440 Université Laval, Quebec City, Quebec G1V 0A6, Canada; ‡ CUO - Recherche, CHU de Québec Research Center - Université Laval, Quebec City, Quebec G1S 4L8, Canada

## Abstract

The S100B protein plays diverse roles in cellular mechanisms, particularly at the membrane, where it is suggested to exhibit chaperone-like functions, stabilizing the conformation of the p53 protein and facilitating its nuclear translocation and accumulation. However, the membrane binding ability of the S100B protein and the parameters affecting these interactions remain unclear. This study thus explores the membrane binding of S100B using the Langmuir monolayer model coupled with surface tensiometry in order to better understand its role where lipids are involved in its environment. S100B was overexpressed in E. coli and purified by affinity chromatography. The binding parameters and kinetics of purified S100B were measured using surface tensiometry in the presence and absence of calcium ions. S100B preferentially interacts with unsaturated phospholipids, particularly those with PE and PS head groups. Also, the presence of calcium ions enhances these interactions by inducing conformational changes in the S100B structure. The binding kinetics revealed increased interaction with unsaturated lipids, underscoring the importance of the lipid environment in modulating the S100B function. In addition, the interaction observed in the monomeric form indicates that dimerization is critical for S100B’s effective membrane binding. These findings provide new insights into S100B’s interaction with phospholipids, enhancing our understanding of the factors influencing its membrane behavior.

## Graphical abstract

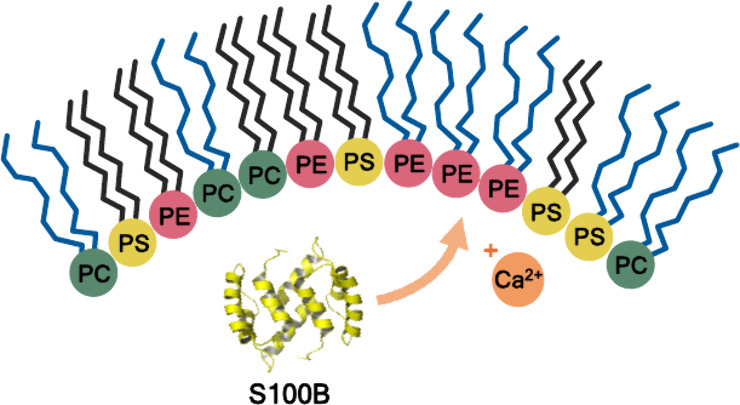

## Introduction

S100B is a protein belonging to the EF-hand protein superfamily^1,2^ and, more specifically, to the S100 protein family, first identified by Moore in 1965.^3^ The S-100 protein was initially discovered as a soluble protein typical of the nervous system; it was later classified as two distinct but similar proteins, S100A1 and S100B. This discovery led to the establishment of the S100 protein family, named due to the ability of the original members to be solubilized in a saturated solution of ammonium sulfate at neutral pH. As of 2024, 25 members of this family have been identified in humans, including S100A1–S100A18, S100B, S100G, S100P, and S100Z, filaggrin, repetin, and trichohyalin.^4^ Some classifications also include “fused gene” proteins such as cornulin,^5^ hornerin,^6^ and filaggrin-2 ^7^ as part of the S100 protein family. The S100 proteins are a subfamily of EF-hand proteins,^1,2^ named after the EF-hand motif found in parvalbumin, composed of two alpha helices (helix E and helix F) connected by a loop that binds a calcium ion.^8^ All S100 proteins, except S100A10 ^9^ and S100A14,^10^ undergo conformational changes upon binding calcium. The S100 protein family is distinct from other EF-hand proteins in several ways: they have different sequences for the two EF-hand motifs within the same protein, they can form both homo- and heterodimeric conformations,^11^ and they are specifically expressed in various tissues and cells.^12^ Notably, S100 proteins are expressed only in vertebrates.^13^ S100B is highly expressed in nervous tissues,^14–16^ soft and adipose tissues,^17^ heart,^18^ skin,^19^ and colon.^20^ It is also found in various cell types, including glial cells,^21,22^ immune cells,^23,24^ melanocytes,^25^ and some cancer cell lines.^26^ S100B is involved in various crucial cellular mechanisms and functions, especially at the membrane.^13^ S100B is increasingly studied in cancer research (see for a review on the subject of S100 proteins in cancer^27^) and proposed as a biomarker for diagnosis or prognosis of breast,^28^ lung,^29^ ovarian,^30^ and colorectal cancer,^31^ among others, brain injuries,^32^ and pigmented skin lesions,^33^ as well as a potential therapeutic target.^34,35^

S100B is unique among S100 proteins for its high-affinity binding to the p53 dimer and protomer,^36^ suggesting a role in stabilizing p53 in its native conformation before nuclear or mitochondrial translocation.^37^ This interaction promotes nuclear accumulation of p53 and activation of its growth suppression and apoptotic functions.^38,39^ Additionally, S100B has been shown to inhibit the transcriptional activity of p53 linked to apoptosis,^40^ potentially acting as a chaperone to facilitate proper folding and nuclear translocation of p53. Similar chaperone-like functions have been proposed for other proteins and S100 family members.^41–46^

Previous studies have demonstrated that other S100 proteins interact with membranes, using a variety of membrane models such as lipid monolayers, multilayer vesicles, and live cell membranes.^47–49^ Additionally, the interaction of S100B with membranes and phospholipids—ranging from cardiolipin vesicles studied through fluorescence, circular dichroism and electron spin resonance spectroscopy, turbidity measurements and electron microscopy, to synaptosomal particulate fractions whose bilayer properties were perturbed by S100 binding as shown by spin labeling^50–52^ has previously been demonstrated using both artificial and natural membrane models. These findings highlight a potential functional role for S100B in modulating membrane structure and dynamics under physiological or stress conditions.

This research investigates the membrane binding of purified S100B using Langmuir monolayer models and surface tensiometry analysis. Given that S100B undergoes conformational changes upon binding calcium, experiments were conducted in both the absence and presence of calcium ions. The Langmuir monolayer model, which allows the investigation of one leaflet at a time from an asymmetric membrane, provides a controlled platform for detailed analysis of lipid–protein interactions at the membrane interface,^53^ providing control over experimental parameters such as buffer conditions, lipid physical state, and surface pressure.^54^

The results presented offer insights into the membrane behavior of S100B in the presence of multiple lipids as well as the influence of calcium ions on these interactions. This study focuses on the interactions between S100B and specific phospholipids at the molecular level, aiming to capture intrinsic properties of the protein that could underlie its broader capacity for membrane association. This approach, therefore, allows us to dissect how calcium binding and lipid composition modulate S100B’s membrane affinity and thermodynamic behavior, which may inform future studies investigating its interactions with cellular membranes in more complex contexts.

## Materials and Methods

### Materials

Deionized water was from a Barnstead Nanopure system (Barnstead, Dubuque, IA, USA), with resistivity and surface tension of 18.2 MΩ·cm and 72 mN/m, respectively. E. coli BL21-CodonPlus (DE3)-RIL Competent cells and XL10-Gold were procured from Agilent Technologies (Santa Clara, CA, USA). Chemicals such as tryptone, yeast extract, ampicillin sodium salt, isopropyl β-D-1-thiogalactopyranoside (IPTG), glycerol, Tris base, ethylenediaminetetraacetic acid (EDTA) disodium salt dihydrate, dithiothreitol (DTT), sodium dodecyl sulfate (SDS), glycine, ammonium persulfate (APS), SeeBlue Pre-Stained Protein Standard, and 2,6-di*tert*-butyl-4-methylphenol were acquired from Fisher Scientific (Hampton, NH, USA). Additionally, NaCl, KCl, lysozyme, anhydrous Na_2_HPO_4_, KH_2_PO_4_, glacial acetic acid, and hydrochloric acid were from VWR International (Radnor, PA, USA). Anhydrous d-glucose, bromophenol blue, and Coomassie brilliant blue R-250 were obtained from Bio Basic (Toronto, ON, Canada). Bio-Rad Laboratories (Berkeley, CA, USA) supplied 30% Acrylamide/Bis solution and 2-mercaptoethanol (14.2 M), while 100% ethanol was procured from Greenfield Global (Toronto, ON, Canada). The anion exchange chromatography column, HiTrap Q XL (5 mL), was obtained from Cytiva (Marlborough, MA, USA). High-performance liquid chromatography-grade chloroform and methanol were from Laboratoire Mat (Quebec, QC, Canada). Phospholipids, including 1,2-dimyristoyl-*sn-glycero*-3-phosphocholine (DMPC), 1,2-dipalmitoyl-*sn-glycero*-3-phosphoethanolamine (DPPE), 1,2-dipalmitoyl-*sn*-glycero-3-phospho-l-serine (sodium salt) (DPPS), 1,2-dipalmitoyl-*sn-glycero*-3-phosphocholine (DPPC), 1,2-distearoyl-*sn-glycero*-3-phosphoethanolamine (DSPE), 1,2-distearoyl-*sn-glycero*-3-phospho-l-serine (sodium salt) (DSPS), 1,2-distearoyl-*sn-glycero*-3-phosphocholine (DSPC), 1,2-dioleoyl-*sn-glycero*-3-phosphoethanolamine (DOPE), 1,2-dioleoyl-*sn-glycero*-3-phospho-l-serine (sodium salt) (DOPS), 1,2-dioleoyl-*sn-glycero*-3-phosphocholine (DOPC), 1,2-dilinoleoyl-*sn-glycero*-3-phosphocholine (DLPC), 1,2-diarachidoyl-*sn-glycero*-3-phosphocholine (DAPC), 1,2-didocosahexaenoyl-*sn-glycero*-3-phosphoethanolamine (DDPE), 1,2-didocosahexaenoyl-*sn-glycero*-3-phospho-l-serine (sodium salt) (DDPS), and 1,2-didocosahexaenoyl-*sn-glycero*-3-phosphocholine (DDPC), were purchased from MilliporeSigma (Burlington, MA, USA) with a purity exceeding 99%. Lipid solutions were prepared in chloroform, with the exception of DSPS which was dissolved in a mix of chloroform:methanol:water (65:25:4 v:v:v) at concentrations ranging from 0.1 to 0.2 mg/mL. To prevent oxidation, 2,6-di*tert*-butyl-4-methylphenol (5 μg/mL) was incorporated into the unsaturated lipid solutions as an antioxidant, and these were carefully shielded under an argon atmosphere.^55^ A storage temperature of −20 °C was selected for the preservation of all of the lipid samples and solutions.

### Methods

#### S100B Overexpression and Purification

The S100B protein employed in our study, with a molecular weight of 10 713 Da according to its UniProt Knowledge Base sequence (P04271), originates from Homo sapiens (Human). The S100B gene, harbored in the pETDuet-1 vector, was introduced into E. coli, where it underwent transformation, subsequent overexpression, and purification through anion exchange chromatography. The comprehensive methodology for these different steps has been previously documented.^56^ Briefly, S100B was overexpressed in E. coli BL21-Codon Plus (DE3)-RIL. Bacterial cultures in 1 L of LB ampicillin medium were cultured at 37 °C and 250 rpm until reaching an optical density at 600 nm (O.D. 600 nm) of 0.7. Overexpression was initiated by adding 10 mL of 100 mM isopropyl β-D-1-thiogalactopyranoside (IPTG) into the cultures, maintaining the incubation at 37 °C and 250 rpm for 4 h. Bacterial cultures were then centrifuged at 10,000*g* for 30 min at 4 °C, retaining only the cell pellet. Cell lysis was accomplished with lysozyme in a Tris 50 mM, DTT 5 mM, and EDTA 5 mM buffer (pH 7), followed by three cycles of freeze–thaw and sonication (3 min of discontinuous sonication: 5 s “on”, 5 s “off” for 150 s followed by 30 s of continuous sonication). The lysed cells underwent centrifugation at 10,000*g* for 30 min at 4 °C, and the cell pellet was resuspended in an equal volume of the aforementioned Tris buffer. Initial removal of contaminants was performed through ammonium sulfate purification. Ammonium sulfate salt was added to the resuspended pellet, reaching a saturating concentration while maintaining the solution at 4 °C and a pH of 7. The solution was then centrifuged at 10,000*g* for 60 min at 4 °C. Subsequently, the pH of the supernatant was lowered to 4 and centrifuged again at 10,000*g* for 60 min at 4 °C. The resuspended pellet was loaded onto a HiTrap Q XL (5 mL) anion exchange column pre-equilibrated with 25 mM Tris–HCl, 1 mM DTT, and 1 mM EDTA (pH 8.0) using an AKTA FPLC (GE Healthcare) at a flow rate of 0.5 mL/min. After column washing at 2 mL/min for 10 column volumes, the protein was eluted using a 30 min linear gradient of the identical buffer containing 1 M NaCl (flow rate of 1 mL/min). Fractions containing purified S100B were identified by SDS-PAGE. The SDS-PAGE gel (16%) was scanned, and ImageJ analysis determined the purity of S100B. Evaluation of purity using ImageJ revealed that S100B purity exceeded 95% (refer to Supporting Information, Figure S1, for the Coomassie-stained gel image of purified S100B). To confirm the identity of the S100B protein, LC/MS-MS analysis of the SDS-PAGE gel was conducted, resulting in a 100% probability match to S100B. Circular dichroism analysis of the pure S100B protein at different temperatures and durations demonstrated its stability over a range of conditions, including 4, 20, −20, or −80 °C, for a minimum of 60 days (refer to Supporting Information, Figure S2, for circular dichroism spectra of S100B). Consequently, S100B exhibits stability during analysis at 20 °C and is stored at 4 °C. The monomeric form of S100B (low-salt S100B) was obtained using 1 mg/mL protein solutions and extensive dialysis against deionized water at pH 7 to ensure extensive desalting of the samples.^57^ The obtention of monomeric S100B was verified with native gel migration (Figure S3).

#### Surface Pressure Measurements

The surface pressure, essential for calculating protein binding parameters, was determined by using the Wilhelmy method. A DeltaPi4 microtensiometer (Kibron Inc., Helsinki, Finland) and a Teflon trough (volume: 1000 μL; diameter: 18 mm; depth: 5 mm) were employed for surface pressure (Π) measurements. Humidity control was maintained in a Plexiglas box, and the experimental temperature was held constant at 20 ± 1 °C. The trough’s subphase consisted of 1000 μL of buffer containing 200 mM Tris and 100 mM NaCl at pH 7 for experiments conducted without calcium. For measurements in the presence of calcium, 5 mM CaCl_2_ was added to the buffer. To establish the saturating (equilibrium) concentration, varying volumes of S100B were introduced beneath the subphase surface with a syringe. The determined saturating concentration was 1 μM (equivalent to 0.011 mg/mL), corresponding to an equilibrium surface pressure of 21.6 mN/m without calcium and 16.7 mN/m with calcium (Figure 1). This concentration marked the initiation of saturation of the interface by the protein and was employed for all subsequent experiments. First, a few microliters of a specified phospholipid solution were initially deposited onto the subphase. The solvent was allowed to spread and evaporate, and the phospholipid film reached surface pressure equilibrium, representing the initial surface pressure of the lipid film (Π_i_). The time required for this process was influenced by the lipid type, spreading volume, and initial surface pressure. S100B was then injected beneath the lipid monolayer to achieve a concentration above the saturating concentration of 0.02 mg/mL (the stock solution concentration was 2 mg/mL). The interaction between S100B and the phospholipid monolayer was monitored by measuring the surface pressure until the equilibrium surface pressure (Π_e_) was reached. The surface pressure variation (ΔΠ) corresponds to Π_e_–Π_i_ and is indicative of the presence of the protein at the interface. After each experiment, the Teflon trough was meticulously cleaned by being rinsed three times with distilled water, followed by three rinses with ethanol. Subsequently, a clean cotton swab was used to carefully wipe the cuvette systematically: clockwise, top to bottom, left to right, diagonally, and finally counterclockwise.

**Figure 1:**
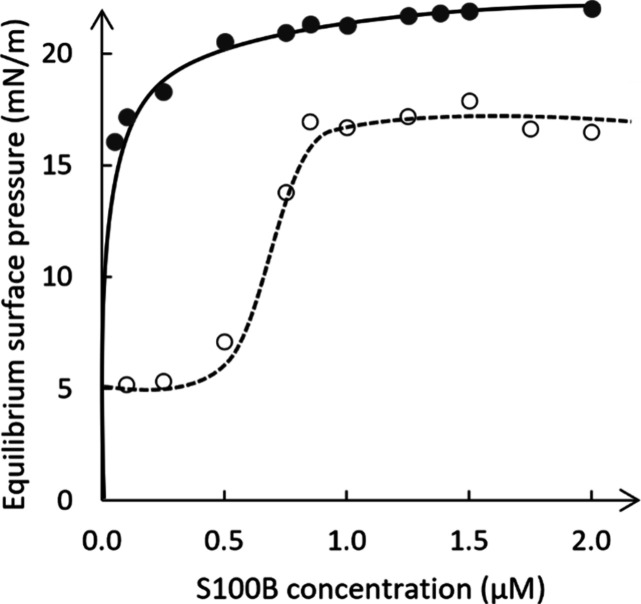
Determination of the S100B saturating concentration without (black dots) and with calcium ions (white dots). The lines are used as a guide to the eye.

#### Determination of the Binding Parameters

The determination of binding parameters and uncertainty calculation has already been described^54,58^ and used for several calcium-binding proteins.^59,60^ Briefly, the surface pressure variation (ΔΠ) resulting from the injection of S100B was plotted against Π_i_ and subjected to linear regression analysis (see Figure 2 for the evolution of Π over time and its insert for the linear regression analysis of ΔΠ as a function of Π_i_) with a minimum of 10 data points being used. The maximum insertion pressure (MIP) was determined at the point of intersection with the *x*-axis, and its uncertainty was computed based on the covariance of the experimental data in relation to the regression. The synergy (a theoretical depiction of this concept is provided in Figure S4 of the Supporting Information) is defined as 1 plus the slope, and its uncertainty was calculated as (σ­(Π_e_) × (1 – *r* ^2^)^1/2^)/(σ­(Π_i_) × (*n* – 2)^1/2^), where σ represents the standard deviation, *r* is the correlation coefficient, and *n* is the number of data points.^61^ All calculations can be performed using online software available at http://www.crchudequebec.ulaval.ca/BindingParametersCalculator.

**Figure 2:**
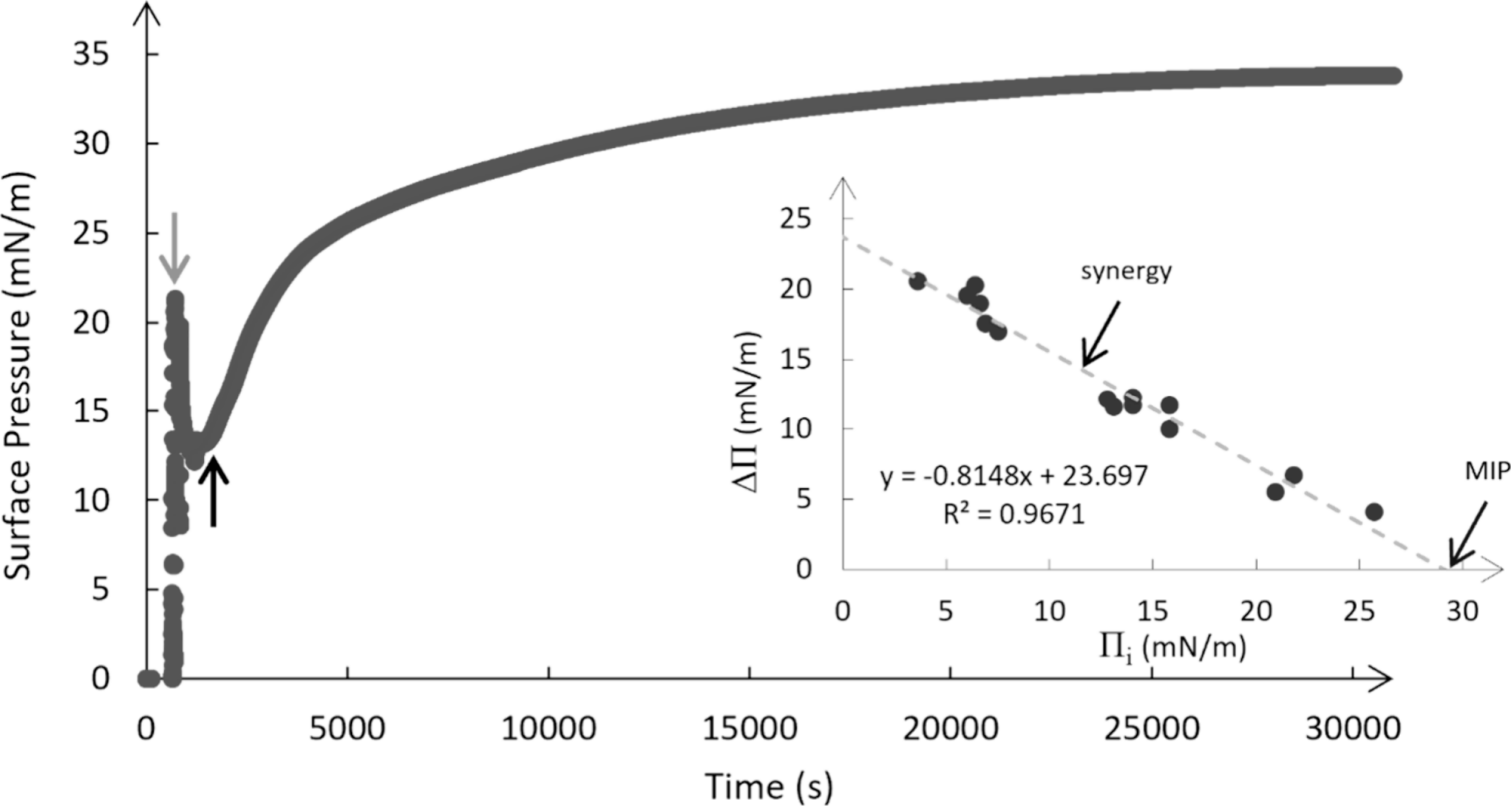
Kinetics of surface pressure over time during the interaction between S100B and a DSPE monolayer without calcium ions: At the start of the experiment, the surface pressure of the aqueous buffer is set to zero. DSPE is then deposited onto the buffer surface, resulting in the first noticeable increase in pressure (starting at approximately 600 s indicated by the first arrow in gray) until stabilization is achieved and the initial pressure of the lipid film (Π_i_) is reached around 1200 s (second arrow in black). S100B is then injected into the subphase, leading to a gradual increase in surface pressure due to the protein’s binding kinetics. This increase continues until a second equilibrium (Π_e_) is reached, fully stabilized around 29,000 s; inset: linear regression of ΔΠ as a function of Π_i_ for the determination of the binding parameters where each point in the linear regression corresponds to one S100B binding kinetic experiment with DSPE.

#### Surface Pressure Kinetics Analysis

The kinetics of surface pressure were investigated by observing the surface pressure variation (ΔΠ) subsequent to the injection of proteins over time. The kinetics were modeled using the stretched exponential equation tailored for surface pressure measurements, as proposed by Pitcher et al., Π_*t*_ = Π_e_ – Π_i_e^–(*kt*)β^.^62^ In this equation, Π_*t*_ represents the surface pressure of the monolayer at time *t*, *k* is the rate coefficient, *t* is time, and β is the exponential scaling factor, initially set to 0.001 to optimize the fitting accuracy of the adsorption isotherms.

#### Protein Membrane Binding Simulations

The interaction between the S100B protein and lipid membranes was modeled using the PPM server from the OPM database. The PPM server predicts the orientation and positioning of membrane-associated proteins based on their three-dimensional structure and physicochemical properties, including hydrophobicity and electrostatic potential. For these simulations, the crystal structure of S100B (PDB IDs: 1PRU for the calcium-free form and 1UWO for the calcium-bound form) was uploaded to the PPM server. The membrane in the model was composed of DOPC, which was consistent with the experimental conditions. The server computes the optimal positioning of the protein relative to the membrane, determining insertion depth and orientation by considering hydrophobic mismatches and electrostatic interactions with lipid headgroups.^63^ This method enabled us to predict the most favorable binding conformation of S100B on the lipid membrane under these conditions, offering a detailed view of its membrane association.

## Results and Discussion

To obtain information about the molecular interaction between S100B and membrane lipids, we investigated the binding of S100B to phospholipid monolayers using surface tensiometry. Phospholipids consist of a hydrophilic polar headgroup, which can be either negatively charged or zwitterionic, and a hydrophobic tail comprising two acyl chains that may be saturated or unsaturated. These distinct characteristics can significantly influence their interactions with proteins.^64,65^ For instance, phospholipids with a negatively charged polar headgroup, such as phospho-l-serine, are predisposed to interact with positively charged proteins through electrostatic interactions. Additionally, size plays a crucial role in lipid–protein interactions; despite both phosphocholine and phosphoethanolamine being zwitterionic, the former has a larger steric hindrance, impacting both the polar headgroup and chain packing constraints. Consequently, lipid–protein interactions may be altered, especially when a protein is inserted into the membrane. The physical state and lipid phase can also be influenced by the presence of unsaturated bonds in the acyl chains, affecting membrane organization and consequently lipid–protein interactions. In order to assess the influence of lipid types, calcium ions, and dimerization, experiments were carried out with 12 different lipids, with and without calcium ions (as shown in Figure S5, calcium by itself has no detectable effect on phospholipid monolayers), and also with a monomeric form of S100B, as detailed in the following three sections.

### Influence of the Nature of Phospholipids on the S100B Interaction

#### Choice of Phospholipids and Meaning of the Binding Parameters

Twelve phospholipids, each with different combinations of polar head groups and acyl chains, were employed in the Langmuir monolayer model membrane. Six of these phospholipids had saturated acyl chains, including DPPE, DPPS, and DPPC (DP for diC16:0 associated with the three polar head groups) and DSPE, DSPS, and DSPC (DS for diC18:0 associated with the three polar head groups). The remaining six phospholipids were unsaturated, with DOPE, DOPS, and DOPC (DO for diC18:1 associated with the three polar head groups) and DDPE, DDPS, and DDPC (DD for diC22:6 associated with the three polar head groups).

One of the measured binding parameters in these experiments is the maximum insertion pressure (MIP), representing the pressure from which a protein can no longer insert into a lipid membrane (Figure 2). The second binding parameter examined is synergy, indicating the type of interaction occurring between the protein and the lipid monolayer. The synergy parameter in protein–membrane interactions quantifies how cooperative or antagonistic the interactions are between a protein and a lipid monolayer, offering critical insight into the nature of the binding. It provides a numerical measure that captures whether the interaction is favorable (synergistic) or unfavorable (antagonistic), and can therefore distinguish between mere surface contact and significant binding.^61^ Considering that the synergy value is defined as 1 plus the slope, a positive synergy value indicates that the protein–lipid interaction is cooperative, meaning that protein binding to the membrane becomes increasingly favorable with higher lipid concentrations at the interface. In this case, the protein is attracted to the membrane, likely due to several factors such as hydrophobic interactions between the protein and the lipid chains or electrostatic complementarity, where charged regions of the protein are drawn to oppositely charged lipid headgroups, promoting binding. Thus, the synergy parameter highlights how local variations in membrane composition can create an optimal environment for protein interaction. On the contrary, a negative synergy value signals that repulsion exists between the protein and the lipid monolayer, which might occur due to electrostatic repulsion, where charges on the protein and membrane repel each other, or rigidity mismatches between the protein and lipid environment that prevent effective binding. This repulsion reflects a physical barrier that restricts the protein from interacting with the membrane, suggesting that specific lipid compositions can hinder protein binding. The synergy and MIP values for S100B with the 12 studied lipids were gathered in Figure 3 (the numerical values of MIP and synergy are also listed in Tables S1 and S2, respectively), and the influence of several parameters (head groups, saturations, kinetics, etc.) was described in the following sections. Regarding MIP values, it is also valuable to compare the experimentally obtained MIP values to the estimated value of the lateral pressure of the cell membrane, which has been estimated to be around 30 mN/m.^66–69^ This comparison helps determine whether the protein’s insertion pressure is sufficient to allow stable interaction or integration with the lipid monolayer under conditions similar to those in cellular environments.

**Figure 3:**
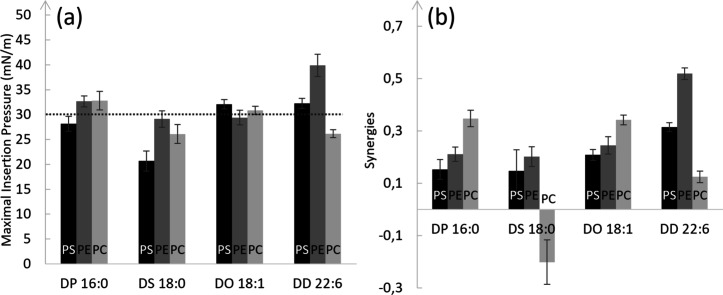
Comparison of MIP (a) and synergy (b) values for S100B in the absence of calcium.

#### Interaction of S100B with PE Lipids

Starting with lipids containing a phosphoethanolamine (PE) headgroup, the linear regression of ΔΠ as a function of Π_i_ (used to determine their binding parameters) is shown in Figure S6, DSPE had a slightly lower MIP value (29.1 ± 1.6 mN/m) compared to DPPE (32.7 ± 1.1 mN/m), although their synergy values were similar (0.20 ± 0.04 for DSPE and 0.21 ± 0.03 for DPPE). When comparing the saturated phospholipid DSPE with the monounsaturated DOPE, both having the same number of carbon atoms in their acyl chain length, the MIP and synergy values were comparable (29.4 ± 1.5 mN/m and 0.24 ± 0.03 for DOPE and 29.1 ± 1.6 mN/m and 0.20 ± 0.04 for DSPE). In contrast, the polyunsaturated DDPE showed significantly higher MIP (39.93 ± 2.2 mN/m) and synergy (0.52 ± 0.02) values than all the other PE lipids, suggesting that S100B prefers interacting with PE unsaturated lipids, and this interaction is enhanced with a higher number of unsaturations in the acyl chain. In addition, taking uncertainty into account, all PE phospholipids display MIP values reaching or exceeding 30 mN/m, which, as previously mentioned, is the estimated lateral pressure of cell membranes. These values suggest that S100B would be capable of interacting with bilayer domains composed of these lipids under physiological conditions. Interestingly, the interaction of S100B with DDPE exhibits the highest MIP and synergy values observed overall.

#### Interaction of S100B with PC Lipids

For the lipids with a phosphocholine (PC) polar headgroup, the linear regression of ΔΠ as a function of Πi (used to determine their binding parameters) is shown in Figure S7. Saturated DSPC had a lower MIP value (26.1 ± 1.9 mN/m) than DPPC (32.8 ± 1.9 mN/m). The synergy obtained with DSPC is notably lower (−0.20 ± 0.08) compared with DPPC (0.35 ± 0.03). DSPC is the only studied lipid leading to a negative synergy, meaning that S100B has very little chance of interacting with a part of the membrane containing mainly DSPC. The presence of one unsaturation increased both the MIP and synergy values: DOPC (30.9 ± 0.8, 0.34 ± 0.02 mN/m) vs DSPC (26.1 ± 1.9, −0.20 ± 0.08 mN/m). However, increasing the degree of unsaturation with DDPC lowered these values (26.2 ± 0.8 and 0.12 ± 0.02 mN/m), unlike what was observed with the PE lipids. Also, it is worth noting that DSPC and DDPC exhibit MIP values below 30 mN/m, suggesting that S100B would likely be unable to interact with bilayer domains composed of these lipids under physiological conditions. These data suggest that S100B preferentially interacts with PC phospholipids composed of DP and DO acyl chains, and increasing the number of carbons (to DS and DD, respectively) seems to hinder this interaction. Furthermore, Figure S8, in the Supporting Information, illustrates the relationship between synergy and MIP values for both saturated (Figure S8a) and unsaturated (Figure S8c) PC phospholipids in their calcium-free interaction with S100B. As shown in Figure S8a, as MIP values increase, synergy values also rise, suggesting that greater surface pressure (reflected by higher MIP values) leads to more favorable and synergistic binding (indicated by higher positive synergy values). This observation may therefore explain the low synergy value of DSPC in the calcium-free state, as DSPC exhibits the lowest MIP value. A similar trend is observed in Figure S8c for unsaturated PC phospholipids, where increased MIP values correlate with higher synergy values observed in the absence of calcium.

#### Interaction of S100B with PS Lipids

For the lipids containing an anionic phosphoserine (PS) polar headgroup, the linear regression of ΔΠ as a function of Πi (used to determine their binding parameters) is shown in Figure S9, DSPS had a lower MIP value (20.7 ± 2.0 mN/m) than DPPS (28.2 ± 1.5 mN/m), with similar synergy values (0.15 ± 0.04 for both). Comparing DSPS with the monounsaturated DOPS, both having the same acyl chain length, DOPS had significantly higher MIP (32.1 ± 0.9 mN/m) and synergy (0.21 ± 0.02) values than DSPS (20.7 ± 2.0 and 0.15 ± 0.08 mN/m). The polyunsaturated DDPS had approximately the same MIP value (32.3 ± 1.0 mN/m) as DOPS, but a higher synergy value (0.31 ± 0.02 vs 0.21 ± 0.02). Notably, among the PS phospholipids, only DSPS shows an MIP value below 30 mN/m, suggesting that S100B would not interact with bilayers composed of DSPS under physiological conditions. These data suggest that S100B prefers interacting with unsaturated acyl chains in the PS group, favoring polyunsaturated phospholipids over monounsaturated phospholipids.

#### Influence of the Phospholipid Physical State

The high binding parameter values observed in the presence of unsaturated lipids suggest that S100B interaction increases with higher fluidity, which is associated with the physical state of the phospholipids within the lipid monolayer.^70^ At the experimental temperature of 20 °C, unsaturated phospholipids are in the liquid-expanded (LE) phase, while saturated phospholipids are typically in the LE/liquid-condensed (LC) coexistence region and progressively enter the LC phase with increasing initial surface pressure—this transition occurring at lower initial pressures as the length of the acyl chains increases.^71^ In the LC phase, protein insertion into phospholipid monolayers is more challenging, whereas in the LE phase, S100B insertion is favored, which could possibly be due to hydrophilic interactions with the polar headgroup as well as hydrophobic interactions with the acyl chains. In addition, saturated phospholipids form highly ordered, tightly packed monolayers due to the lack of double bonds in their fatty acyl chains, which restricts their fluidity, in comparison to those containing unsaturations. As such, when a protein embeds itself partially into the lipid headgroup region or deeper into the hydrophobic tails of such monolayers, it could potentially disrupt the tight packing of the saturated acyl chains and could then be responsible for a more disordered, fluid-like state. As a result, lateral pressure increases could not only be responsible for the alteration of membrane stability and fluidity but also could influence phospholipid–protein interactions. Despite the inner core of the S100B homodimer containing hydrophobic residues, several hydrophobic residues on its periphery (as shown in Figure S10) could interact with acyl chains according to the protein orientation.

#### Influence of the Charge

The calculated pI of S100B is 4.54, indicating a strong negative charge (−28.1) at pH 7, which could disrupt interactions with PS head groups. However, the negative charges are mainly on both sides of the hydrophobic cleft of the S100B homodimer, possibly allowing interaction with PS with positive charges on the outer surface of S100B, despite potential repulsive forces. This may explain why saturated phospholipids with PS head groups had generally lower MIP values than those with PE and PC head groups, without necessarily observing negative values of synergy.

#### Influence of the Steric Hindrance

Steric hindrance also affects S100B binding, as PE headgroup exhibits a smaller molecular area than PC headgroup, itself being slightly smaller than PS headgroup, mainly due to the charge contribution.^72,73^ For S100B, differences can be noticed with polyunsaturated ones. Hence, for DDPE, DDPS, and DDPC, the highest MIP and synergy values were observed with the PE headgroup, which has the smallest molecular area. The presence of PE seems to favor S100B binding, allowing easier access to the acyl chains. The presence of the PS headgroup also leads to high values of MIP and synergy, compared to PC. Indeed, the fluidity of DD acyl chains offers space around the PS headgroup and allows the protein to benefit from the potential electrostatic interactions with the negatively charged PS.

#### Interaction of S100B with Saturated Phospholipids

For saturated phospholipids with identical acyl chain types, the MIP values were higher for phospholipids with PE or PC polar head groups (DPPE: 32.7 ± 1.1, DSPE: 29.1 ± 1.6, DPPC: 32.8 ± 1.9, DSPC: 26.1 ± 1.9 mN/m) compared to those with a PS polar headgroup (DPPS: 28.2 ± 1.5, DSPS: 20.7 ± 2.0 mN/m). Saturated acyl chains seem to enhance S100B interaction more effectively with PE and PC head groups than with PS. Synergy values, however, did not follow the same trend. Both PE and PS head groups showed similar synergy values (DPPE: 0.21 ± 0.03, DSPE: 0.20 ± 0.04, DPPS: 0.15 ± 0.04, DSPS: 0.15 ± 0.08), while DPPC exhibited a higher synergy value (0.35 ± 0.03) and DSPC a lower one (−0.20 ± 0.08). All the MIP and synergy values observed with DP and DS lipids are relatively low, suggesting that S100B will not preferentially bind these lipids within the biological membrane. The higher observed synergy for DPPC compared to other studied dipalmitoyl (DP) phospholipids may be attributed to its physical state at experimental temperature (20 °C). While DPPE and DPPS are in a solid-condensed phase under these conditions, DPPC is at the phase transition between the liquid-expanded and solid-condensed states. This more disordered transitional state of DPPC may facilitate a greater interaction with S100B, thereby explaining the enhanced synergy. The synergy between S100B and DSPC is the only negative synergy indicating that the DSPC monolayer appears to be repulsive toward S100B, which would imply that the packing of distearoyl (DS) acyl chains during S100B interaction induces a lipid organization that disrupts the binding of S100B when compared to other acyl chains with the same phosphocholine (PC) polar headgroup.

#### Interaction of S100B with Unsaturated Phospholipids

For the monounsaturated phospholipids, the MIP values for PE, PS, and PC head groups were similar, but synergy values were higher for DOPC compared to DOPS and DOPE. This indicates that S100B interacts with monounsaturated phospholipids regardless of the headgroup but has a stronger physical attraction to DOPC due to its higher synergy value. For phospholipids with a PS polar headgroup, S100B binding is influenced by hydrophilic, hydrophobic, and electrostatic interactions, leading to higher binding parameter values for unsaturated, particularly polyunsaturated, phospholipids compared to saturated ones. Similarly, the higher binding parameter values observed for unsaturated PE phospholipids may be explained by the likelihood that S100B interacts with these lipids primarily through a combination of hydrophilic and hydrophobic interactions, facilitated by the unsaturated acyl chains and the nature of the PE headgroup. Also, multiple double bonds in the DD acyl chains appear to reduce interaction with the PC headgroup compared to DOPC, possibly due to differences in acyl chain region thickness.

#### Binding Kinetics

The kinetics of S100B’s interaction with PE phospholipid monolayers were examined using the stretched exponential equation for surface pressure measurements, revealing a consistent trend with MIP and synergy values (Figure 4a and Table 1). Interaction kinetics decreased with increasing acyl chain length for saturated phospholipids (DPPE: 3398, DSPE: 7349 s^–1^), reflecting their MIP values. Unsaturated phospholipids (DOPE: 1609, DDPE: 79 s^–1^) exhibited faster kinetics, with DDPE showing the fastest rate, in line with its superior MIP and synergy values. Thus, variations in acyl chain length and unsaturation levels, contributing to increased fluidity, correlate with faster binding kinetics.

**Figure 4:**
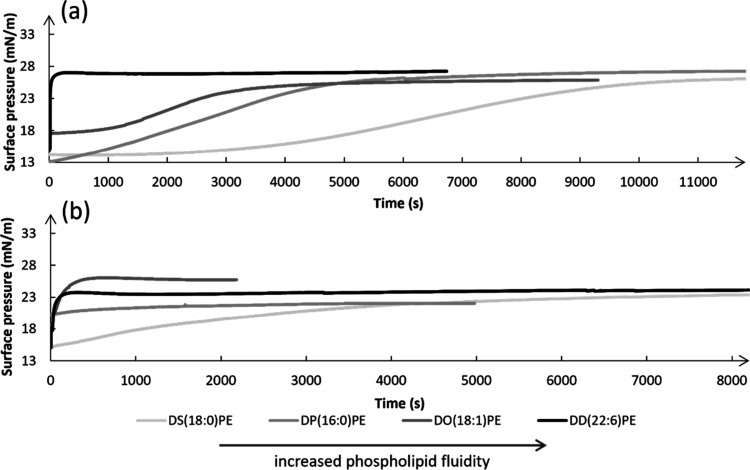
Kinetics of surface pressure over time during the interaction of S100B and PE lipids without (a) and with (b) calcium ions. The curves start at *t* = 0, marking the initiation of binding of S100B to the lipids, which corresponds to the initial surface pressure (Π_i_) of the lipid film in the experiments.

**Table 1:**
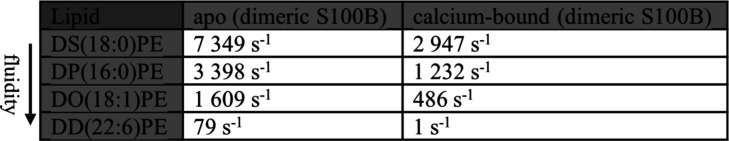
Rate Values of the Binding of Dimeric Apo and Calcium-Bound S100B to PE Phospholipids

To further understand the interactions between phospholipids and S100B, additional studies were conducted in the presence of calcium ions to explore its influence on the S100B membrane interaction.

### Influence of Calcium Ions on the S100B Binding

To further the analysis on S100B membrane behavior, different experiments of binding were performed in the presence and the absence of calcium ions in order to assess the influence of these ions on S100B’s ability to interact with lipids. Given the well-established role of calcium in modulating S100 protein functions,^2^ especially in the context of membrane interactions, this investigation aimed to elucidate how changes in calcium levels might influence the affinity and specificity of S100B for different phospholipids.

#### Influence of Calcium Ions on the Binding Simulation

Simulations using the PPM server were conducted to visualize the interaction of the S100B dimer with a modeled membrane. The PPM server performed the calculation of rotational and translational positions of S100B in membranes by using an optimization of the transfer free energies of S100B from water to the membrane-like environment (defined with the physicochemical and mechanical properties of the chosen lipids). These simulations were performed using the experimental PDB structures in the absence (1PRU) and presence of calcium (1UWO) to assess the impact of the conformational change due to the calcium ions on the interaction between the protein and the modelized membrane. As illustrated in Figure 5, the presence of calcium significantly influences S100B binding to phospholipids, with more residues directly interacting with the membrane (3 residues, His85, Phe88 on helix IV and Glu21′ on helix I′ without calcium, compared to 7 residues Lys5, Asp12 on helix I and His42′, Phe43′, Phe88′, His89′ as wall as Glu91′ on helix IV′ with calcium). Additionally, the S100B conformational change brings α-helix I of one of the monomers in close proximity to the membrane, potentially enhancing the overall interaction and binding of S100B in the presence of calcium. Furthermore, an in-depth investigation of the residue composition of the α-helix I reveals the presence of a hydrophobic face, certainly contributing to the stabilization of the hydrophobic core of the protein (see Figure S11). Additionally, data presented in the Supporting Information, Figure S12, suggest that once S100B is bound to the lipid monolayer, the interaction remains stable and is not easily reversible by the addition or removal of calcium ions under the conditions tested.

**Figure 5:**
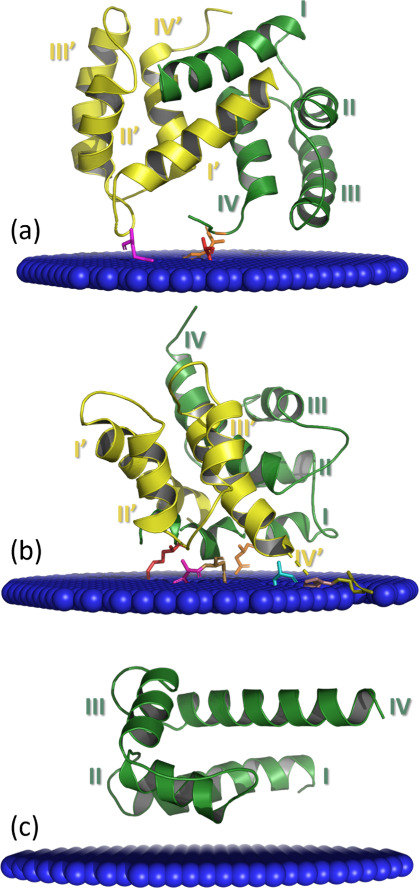
Simulation of the interaction of the dimeric S100B without (a) and with (b) calcium ions as well as the monomeric S100B without calcium (c) with a modeled membrane by the PPM server. Residues observed in (a): E21′ in fushia, H85 in orange and F88 in red. Residues observed in (b): M42′ in fushia, F43′ in brown, F88′ in cyan, H90′ in bronze, E91′ in olive (within the yellow S100B monomer), D12 in orange and K5 in red (within helix 1 of the green S100B monomer).

#### Influence of Calcium Ions on Binding Parameters

In the presence of calcium ions and the associated conformational changes in S100B, an overall beneficial effect on its monolayer binding was observed, as depicted in Figure 6, where MIP values and synergy values in the presence of calcium are reported.

**Figure 6:**
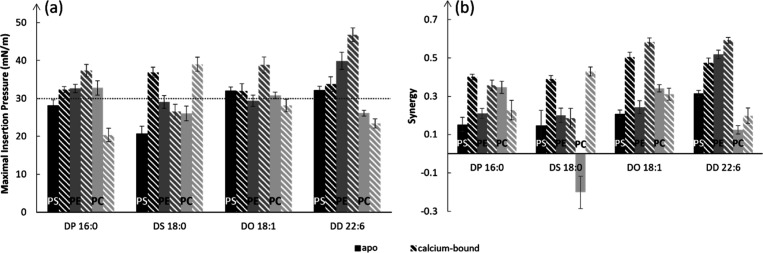
Comparison of MIP (a) and synergies (b) with and without calcium ions.

For 10 out of the 12 studied phospholipids, increased or unchanged binding parameter values were observed. Among the saturated phospholipids, DPPS, DPPE, DSPS and DSPC exhibited increased MIP and synergy values (32.35 ± 0.8 and 0.40 ± 0.01, 37.41 ± 1.6 and 0.36 ± 0.03, 36.90 ± 1.3 and 0.39 ± 0.02, 39.05 ± 1.8 and 0.43 ± 0.02 respectively), while DSPE showed no significant modification (26.6 ± 1.9 and 0.19 ± 0.05), and DPPC displayed decreases in both parameters (20.3 ± 1.8 and 0.23 ± 0.05). Notably, DPPS, DSPS, and DSPC, among the saturated lipids, displayed MIP values above 30 mN/m in the presence of calcium, suggesting that the calcium-induced conformational change in S100B enables an interaction that was not previously possible without calcium, reinforcing its physiological function in calcium-dependent membrane binding. For DSPC in particular, the increase in synergy may be attributed to an improved hydrophobic compatibility between calcium-bound S100B and the lipidic monolayer. Calcium could stabilize the protein–lipid interaction by promoting better alignment between the hydrophobic regions of the lipid tails and the protein, leading to more favorable cooperative binding, where the synergy between the protein and lipids is maximized, thus strengthening the overall binding interaction. Similar to the observations of saturated phospholipids interacting with calcium-free S100B, Figure S8b shows the relationship between synergy and MIP values in the presence of calcium for saturated PC lipids. An increase in MIP values, which corresponds to tighter phospholipid packing and a more ordered monolayer during binding with calcium-bound S100B, is observed. This increase in the MIP values leads to higher synergy values, indicating more cooperative binding and stronger affinity interactions between the phospholipid monolayers and S100B when calcium is present.

A similar trend was observed among unsaturated phospholipids, with five out of seven displaying stable or increasing MIP and synergy values. DOPS exhibited a stable MIP value with an increased synergy value (32.0 ± 2.0 and 0.50 ± 0.03 mN/m), DOPE showed an increase in both parameters (38.8 ± 2.1 and 0.58 ± 0.02), DOPC remained stable in both (28.2 ± 1.6 and 0.31 ± 0.03 mN/m), DDPS had an almost identical MIP value but an increased synergy value (33.8 ± 2.0 and 0.47 ± 0.03 mN/m), and DDPE exhibited increases in both MIP and synergy values (46.8 ± 1.8 and 0.59 ± 0.01 mN/m) while DDPC showed a decreasing MIP value and a slight increase in synergy (23.4 ± 1.3 and 0.20 ± 0.04 mN/m). Only one phospholipid (DOPC) had an MIP value above 30 mN/m in the absence of calcium and below this value in the presence of calcium. Moreover, DDPC, which has a MIP value below 30 mN/m in the absence of calcium, also does not exceed the estimated lateral pressure of the membrane in the presence of calcium. Furthermore, the relationship between synergy and MIP values for unsaturated PC lipids in the presence of calcium shows that, as MIP values increase, synergy values also rise (Figure S8d). In summary, the results obtained in the presence of calcium suggest that calcium-bound conditions promote more favorable synergistic interactions and a stronger binding affinity between the phospholipid monolayers and S100B.

Overall, DSPE and DOPC synergy values remained the same, while all others increased, indicating that the conformational change induced by calcium plays a crucial role in fostering attractive interactions between calcium-bound S100B and membrane phospholipids. Regarding MIP values, the trend was similar to calcium-bound S100B, exhibiting higher MIP values overall, indicating increased affinity toward the phospholipid monolayer. Importantly, the conformational change facilitated a shift in the protein’s binding profile, enabling previously impossible physiological insertions into DPPS, DSPS, and DSPC lipidic membrane domains. These observations suggest a potential physiological function where specific recruitment to DPPS, DSPS, or DSPC domains is necessary at high cellular concentrations of calcium but not during cellular resting states. Conversely, interactions with DPPC domains become physiologically impossible in the presence of calcium. Finally, calcium-bound S100B demonstrates significant affinity toward PS and PE polar headgroup lipids, such as in the absence of calcium, with DDPE exhibiting the highest MIP and synergy values among all studied lipids. Overall, the increased binding observed in the presence of calcium could be attributed to the hydrophobic face on α-helix I (see Figures 3b and S11). Indeed, this hydrophobic region likely contributes to the stabilization of the protein’s hydrophobic core, thereby enhancing the binding ability of the protein with the membrane and thus increasing the MIP values when calcium is present.

#### Influence of Calcium Ions on the Kinetics

Regarding the kinetic aspects of S100B’s interaction with PE phospholipid monolayers in the presence of calcium ions (see Figure 4b), the trend previously observed in the absence of calcium is still observed in the presence of calcium where the kinetics of binding increases with a decrease in the acyl chain length of saturated phospholipids (1232 and 2947 s^–1^ for DPPE and DSPE, respectively). Binding to unsaturated phospholipids, such as DOPE and DDPE, also exhibits higher kinetics than saturated phospholipids (486 and 1 s^–1^, respectively), which is consistent with the MIP values obtained. Furthermore, the kinetics obtained in the presence of calcium are significantly greater than the one observed in the absence of calcium for the same lipids (with more than a 2-fold increase of kinetics for DSPE, almost a 3-fold increase for DPPE, almost a 4-fold increases for DOPE and more than an eighty-fold increase for DDPE) (see Table 1).

### Influence of S100B Dimerization to the Membrane Binding of S100B

The formation of both homo- and heterodimeric S100 proteins indicates that exchange between subunits likely occurs in vivo. Indeed, it has been observed that S100B can interact with S100A1, S100A6, and S100A11 in yeast two-hybrid experiments,^74,75^ suggesting that the subunit interchange of these proteins may be highly dynamic and involved in a potential biological role of S100 monomers. To have a better understanding of the potential role of dimerization in S100B membrane binding, the binding parameters were measured with a monomeric form of S100B. The experiment was performed with the lipid leading to the highest binding parameter values (DDPE) and in the absence of calcium due to the fact that calcium favors dimerization.^76^ The MIP value observed with monomeric S100B (34.33 ± 1.4 mN/m) is lower than that of dimeric S100B (39.9 ± 2.2 mN/m) (Figure 7a). These results align with the PPM server model, showing monomeric S100B (generated with AlphaFold,^77,78^ see Figure S13) situated further from the membrane without any embedded residues, unlike the dimeric form (see Figure 5a vs c). The binding kinetics are also slower for monomeric S100B, with a rate of 4394 s^–1^ compared to 79 s^–1^ for the dimeric form (see Figure 7c). Interestingly, monomeric S100B exhibits a higher binding synergy (0.67) than the dimeric form (0.52) (see Figure 7b). In a physiological context, the higher synergy observed for monomeric S100B is unlikely to compensate for its markedly slower binding kinetics. Despite exhibiting a slightly elevated MIP value as well as a higher synergy compared to the dimeric form, which may suggest some membrane interaction potential, the significantly reduced binding rate could indicate that the monomeric S100B should not be efficiently recruited to membranes under normal conditions. This could support the possible hypothesis that monomeric S100B primarily serves as a dynamic intermediate, readily available for homo- or heterodimer formation, rather than directly engaging in membrane binding.

**Figure 7:**
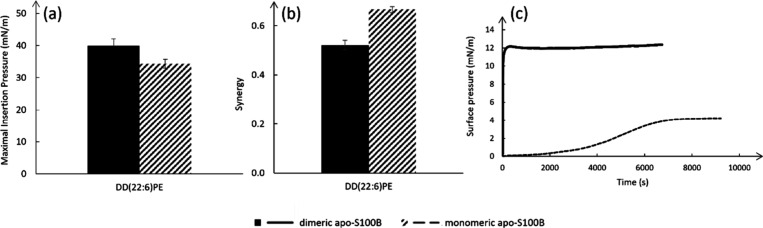
Comparison of the MIP (a), synergy (b) values, and kinetics (c) observed for dimeric and monomeric apo-S100B and a DD(22:6)­PE monolayer with the initial surface pressure of the lipid film set as 0 mN/m for the kinetic representation.

However, the study of monomeric S100B lipidic binding was not extended due to experimental constraints, rendering binding assay results tricky to analyze. Indeed, it seems likely that S100B dimerization occurs at the phospholipid interface, a fact that has already been pointed out in the literature,^76^ causing different experimental equilibrium pressures whose values vary depending on the random amount of interfacial dimerization occurring during the experiment. These preliminary findings highlight the significance of dimerization in facilitating robust membrane binding.

## Conclusions

This study provides significant insights into the membrane binding characteristics of S100B, particularly its interactions with different phospholipid polar head groups and acyl chain compositions. Using Langmuir monolayer models and surface tensiometry analysis, we demonstrated that the interaction strength (MIP values) and binding synergy of S100B are influenced by both the type of polar headgroup and the saturation level of the acyl chains. Our findings reveal that S100B exhibits a stronger interaction with unsaturated phospholipids, especially those with multiple unsaturations in the acyl chains. The polar headgroup also plays a critical role, with PE and PS groups showing, on average, higher PIM values as well as positive synergy values, leading to greater interaction levels compared to PC. This interaction is further enhanced in the presence of calcium, which induces conformational changes in S100B, facilitating its membrane binding. The preferential binding to PE and PS head groups underscores the role of specific lipid environments in modulating S100B function. Moreover, our data indicate that S100B’s membrane interactions are not solely dictated by lipid composition but also by the physical state of the lipid monolayer. The liquid-expanded (LE) state of the lipid monolayer provides a more favorable environment for interaction, with increased binding and kinetics observed for unsaturated lipids in the presence of calcium. Similar patterns of calcium-dependent membrane binding are observed in other EF-hand calcium-binding proteins such as Neuronal Calcium Sensor-1 (NCS1). Indeed, like S100B, NCS1’s interaction with membranes is modulated by calcium, though in a distinct manner. In the absence of calcium, NCS1 exhibits strong monolayer binding, with MIP values exceeding 30 mN/m, indicating possible physiological interaction with membrane domains composed of those phospholipids.^59^ This contrasts with S100B, which shows enhanced monolayer binding in the presence of calcium. In fact, calcium-free NCS1 also shows higher synergy values, reflecting a cooperative interaction, even without calcium. However, once calcium binds to NCS1, a conformational change occurs, exposing negatively charged residues and reducing its affinity for membranes.^59^ This reduction in synergy differs from that of S100B, where calcium binding increases its affinity by inducing structural rearrangements that improve hydrophobic compatibility and potentially alter its electrostatic profile to favor membrane binding. Thus, while both proteins undergo calcium-induced conformational changes, the specific effects on membrane interactions diverge, aligning with their distinct physiological roles. In addition, neurocalcin delta (NCALD), another member of the NCS family, undergoes a calcium-myristoyl switch, a mechanism that exposes its myristoyl group upon calcium binding, thereby enhancing its membrane affinity. This interaction is particularly strong with phospholipids containing PE head groups and saturated acyl chains. The presence of calcium also increases NCALD’s synergy values, indicating a more favorable and cooperative membrane interaction,^60^ comparable to the calcium-enhanced membrane binding observed in S100B. While the underlying mechanisms differ—a myristoyl switch in NCALD versus conformational shifts in S100B—calcium consistently promotes stronger interaction and favors synergistic membrane association in both proteins. These findings place S100B’s calcium-dependent membrane interactions within a broader context of calcium-binding proteins. The modulation of membrane interactions by calcium is a common feature across this family, with distinct outcomes depending on the protein’s structure and physiological role. This underscores the importance of calcium as a regulator of protein–lipid interactions, influencing cellular processes, such as signaling, membrane organization, and protein localization. In this light, our study’s findings not only contribute to understanding S100B’s membrane-binding behavior but also align it with well-established models of calcium-dependent membrane interactions observed in other calcium-sensing proteins like NCS1 and NCALD. Regarding S100B, dimerization also appears to enhance binding and kinetics with lipids, suggesting a comprehensive model where, in a physiological environment, S100B likely prefers to interact with membrane sections composed of unsaturated phospholipids with PE polar head groups. These findings enhance our understanding of S100B’s membrane behavior, suggesting that the modulation of its binding to membranes could significantly impact its functional activities and potentially alter the membrane structure and organization. Although direct membrane-binding assays such as cosedimentation have not been performed, our study offers comprehensive insights into the biophysical interactions between S100B and lipid membranes, highlighting the importance of lipid composition and calcium-mediated conformational changes in modulating these interactions. Future studies should focus on the kinetic aspects of S100B binding and explore its interactions in different cellular contexts to fully elucidate its diverse functional repertoire. Additionally, the chaperone-like function of S100B, supported by its high-affinity binding to p53, suggests a crucial role in stabilizing p53 in its native conformation and promoting its proper folding and nuclear translocation. This function is vital for regulating p53’s growth suppression and apoptotic functions, positioning S100B as a key player in cellular stress responses and tumor suppression. In conclusion, this research advances our understanding of the biophysical interactions between S100B and lipid membranes, contributing to the broader understanding of S100B’s multifunctional roles in cellular physiology and its potential as a therapeutic target in diseases such as cancer and brain injuries.

## Supplementary Material



## Notes

Coomassie-stained 16% SDS-PAGE gel of the purified S100B protein; circular dichroism spectra of S100B stored at different temperatures for 30 days; Coomassie-stained 16% native gel of the dimeric and monomeric S100B; theoretical representation of synergy; influence of calcium ions on the surface pressure of a DAPC monolayers in the absence of protein; linear regression of ΔΠ as a function of Π_i_ for determining the binding parameters of studied phospholipids; correlation plots between synergy and MIP values for PC lipids; S100B PDB structures without and with calcium ions; close-up simulation of the interaction between S100B and calcium ions with a model membrane generated by the PPM server and the helicoidal plot of helix I of S100B; titration of calcium during apoS100B binding to DSPC and titration of EDTA during calcium-bound S100B binding to DSPS; per-residue model confidence score (pLDDT) of monomeric S100B and predicted aligned error of monomeric S100B; MIP values (mN/m) of dimeric apo and calcium-bound S100B as well as monomeric apo-S100B; and synergy values of dimeric apo and calcium-bound S100B (PDF)

## Abbreviations

APS: ammonium persulfate
DTT: dithiothreitol
DMPC: 1,2-dimyristoyl-*sn-glycero*-3-phosphocholine
DPPE: 1,2-dipalmitoyl-*sn-glycero*-3-phosphoethanolamine
DPPS: 1,2-dipalmitoyl-*sn-glycero*-3-phospho-l-serine (sodium salt)
DPPC: 1,2-dipalmitoyl-*sn-glycero*-3-phosphocholine
DSPE: 1,2-distearoyl-*sn-glycero*-3-phosphoethanolamine
DSPS: 1,2-distearoyl-*sn-glycero*-3-phospho-l-serine (sodium salt)
DSPC: 1,2-distearoyl-*sn-glycero*-3-phosphocholine
DOPE: 1,2-dioleoyl-*sn-glycero*-3-phosphoethanolamine
DOPS: 1,2-dioleoyl-*sn-glycero*-3-phospho-l-serine
DOPC: 1,2-dioleoyl-*sn-glycero*-3-phosphocholine
DLPC: 1,2-dilinoleoyl-*sn-glycero*-3-phosphocholine
DAPC: 1,2-diarachidoyl-*sn-glycero*-3-phosphocholine
DDPE: 1,2-didocosahexaenoyl-*sn*-glycero-3-phosphoethanolamine
DDPS: 1,2-didocosahexaenoyl-*sn-glycero*-3-phospho-l-serine (sodium salt)
DDPC: 1,2-didocosahexaenoyl-*sn-glycero*-3-phosphocholine
*E. coli*: *Escherichia coli*
EDTA: ethylenediaminetetraacetic acid disodium salt dihydrate
IPTG: isopropyl β-D-1-thiogalactopyranoside
LB: Luria-Bertan
LC: liquid-condensed
LE: liquid-expanded
PDB: protein data bank
MIP: maximum insertion pressure
SDS-PAGE: sodium dodecyl sulfate-polyacrylamide gel electrophoresis
Π_i_: initial pressure of the lipid film
Π_e_: equilibrium pressure
ΔΠ: pressure differential between the initial and the equilibrium pressure
