# Supplementary material for: Influence of Calcium Ions on the Membrane Binding of S100B Protein
Source: Langmuir. 2025 Aug 29;41(36):24786–98. doi: 10.1021/acs.langmuir.5c03189 (PMC12445344; doi:10.1021/acs.langmuir.5c03189)
Supplement: Supplementary file 1 [file la5c03189_si_001.pdf]

SUPPLEMENTARY INFORMATION

Influence of calcium ions on the membrane binding of S100B protein

Paul Jaouen[1,2], *Élodie Boisselier*[1,2,*]

[1]Department of ophthalmology and Otolaryngology - Head and Neck Surgery, Faculty of medicine, Université Laval, Quebec City, G1V 0A6, Canada;

[2]CUO–Recherche, CHU de Québec Research Center – Université Laval, Quebec City, G1S 4L8, Canada

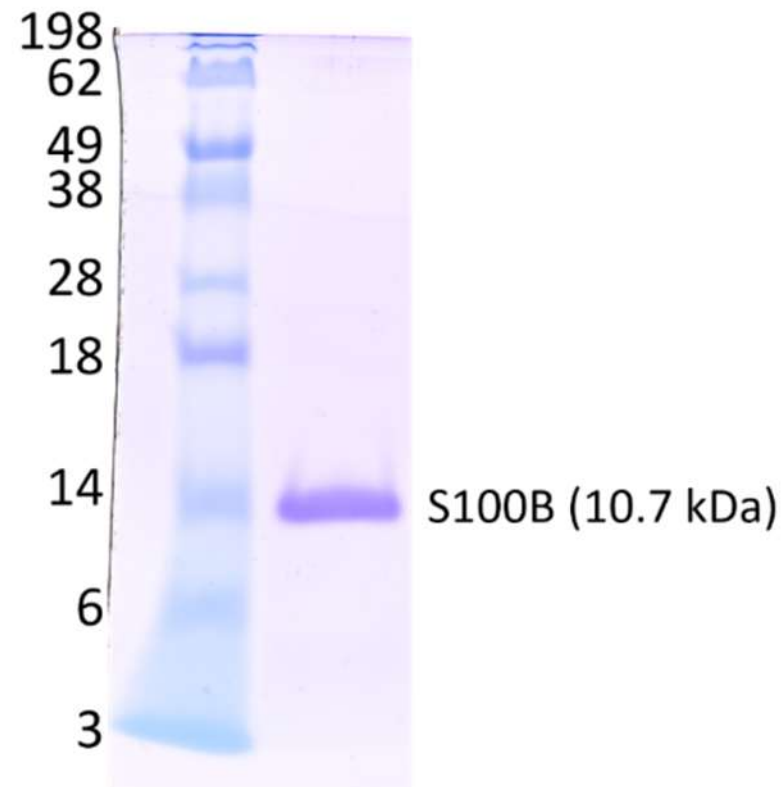

Figure S1: Coomassie-stained 16% SDS-PAGE gel of the purified S100B protein. Numbers on the left indicate the molecular weight in kDa on the proteins within the ladder.

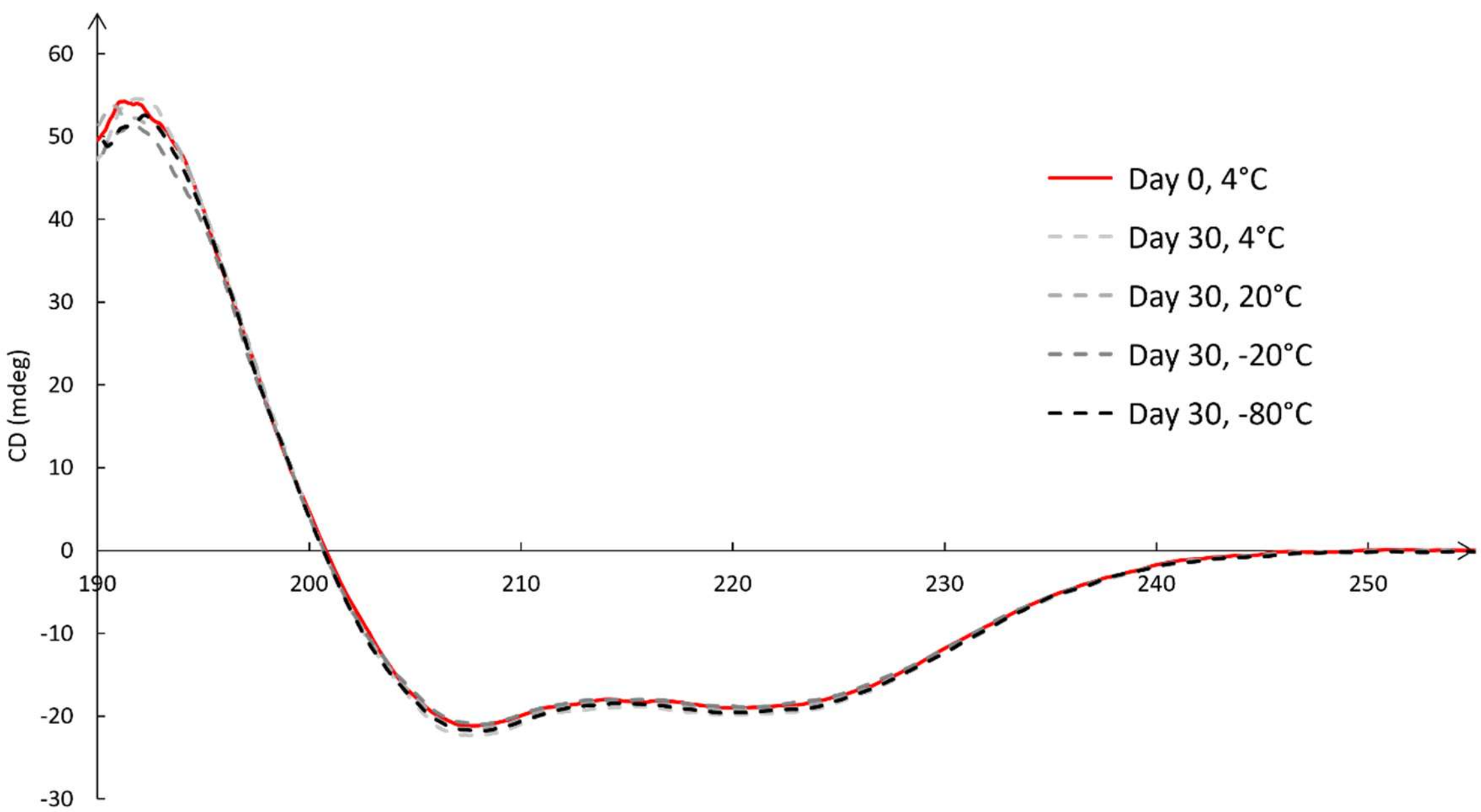

Figure S2: Circular dichroism spectra of S100B stored at different temperatures for 30 days (dashed lines with different gray levels) compared with spectra at 4 °C obtained on day 0 (solid red line).

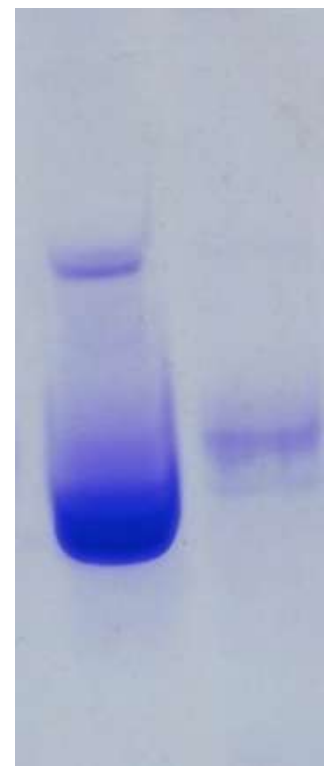

Figure S3: Coomassie-stained 16% native gel of the dimeric and monomeric S100B. The lane on the left is the dimeric S100B in a Tris NaCl buffer, whereas the lane on the right is the same concentration of monomeric S100B in deionized water.

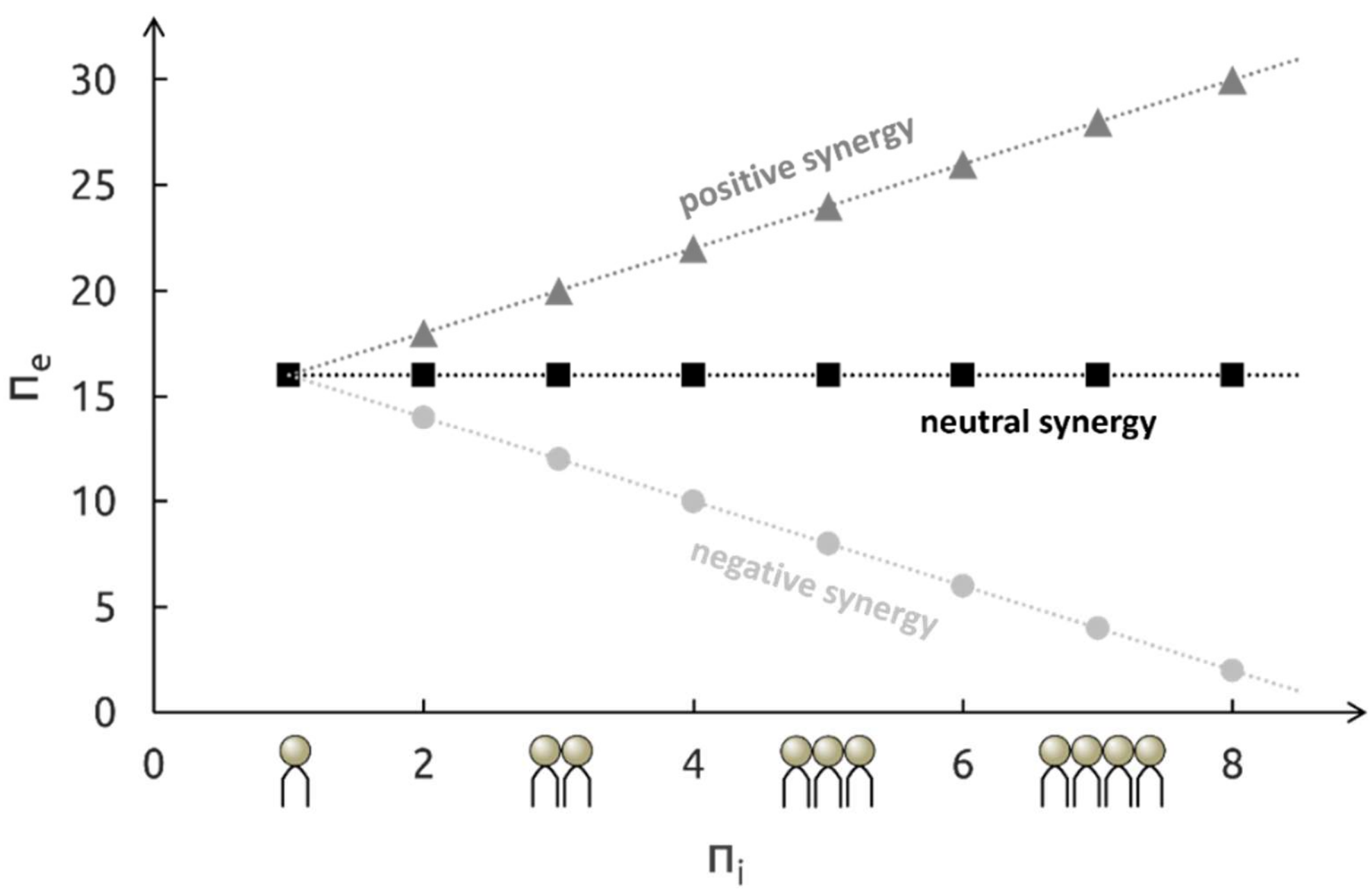

Figure S4. Theoretical representation of synergy, illustrating how equilibrium pressure (Π_e) during protein-membrane interaction changes as a function of initial lipid film pressure (Π_i), which reflects the phospholipid concentration within the monolayer. An increase in Π_e with rising Π_i indicates positive synergy, suggesting a cooperative interaction between the lipid monolayer and the protein. Conversely, a decrease in Π_e as

Π_i increases reflects negative synergy, suggesting a repulsive interaction. When Π_e remains constant despite changes in Π_i, this indicates neutral synergy, with no specific interaction between lipid and protein.

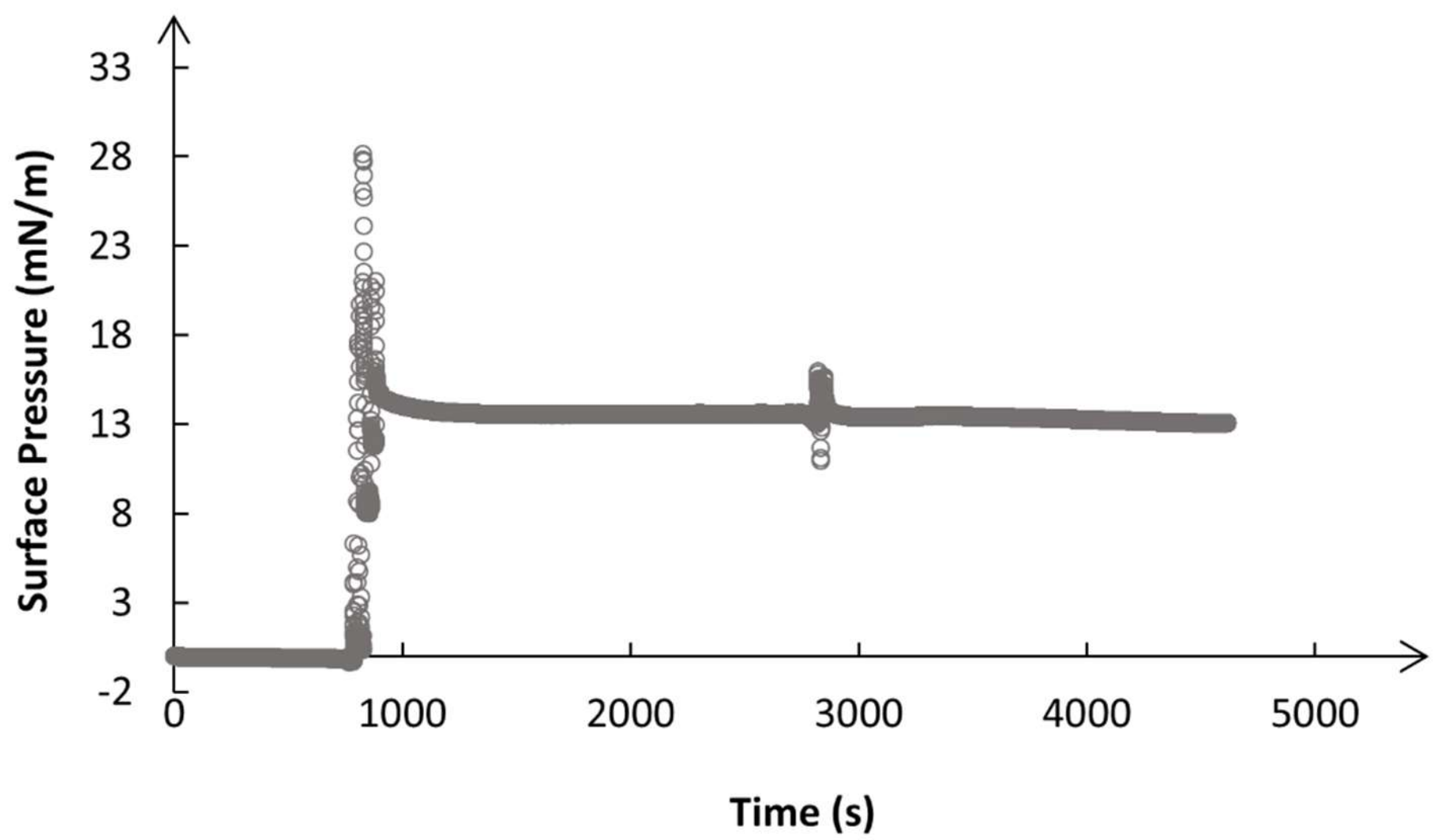

Figure S5. Kinetics of surface pressure over time of a DAPC monolayer in the absence of calcium ions, until equilibrium surface pressure (Πeq) is reached (approximately 2,750 s, reaching 13.6 mN/m). Injection of Ca^{2+} was then performed in the subphase buffer to achieve final calcium concentrations of 20 mM (around 2,850 s). The Ca^{2+} injection resulted in a brief spike in surface pressure, followed by a return to the equilibrium value of approximately 13.5 mN/m, indicating that calcium ions do not intrinsically affect the surface pressure of the phospholipid monolayer.

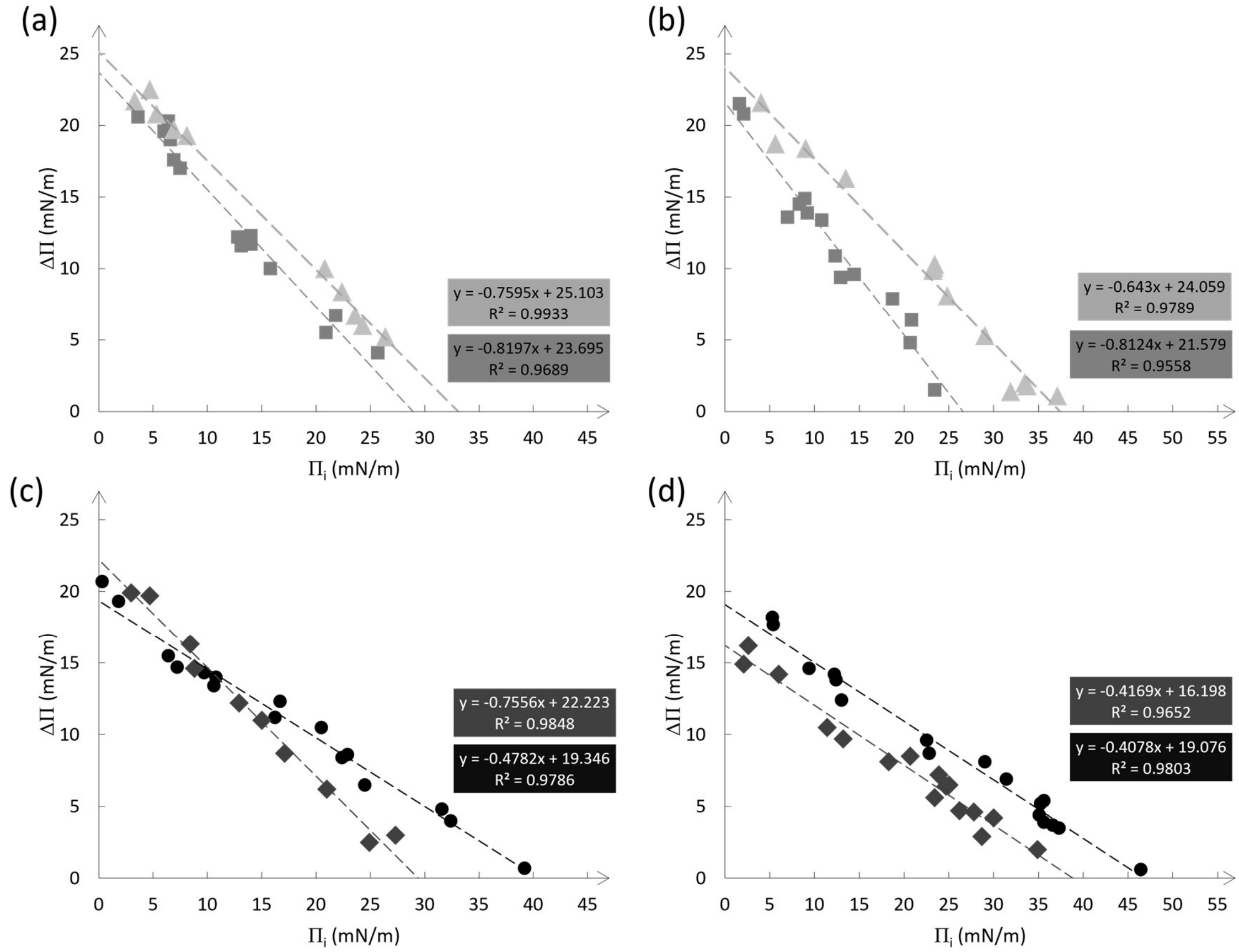

Figure S6. Linear regression of $\Delta\Pi$ as a function of Π_i for determining the binding parameters of PE lipids. Panels (a) and (b) show results for saturated lipids, while panels (c) and (d) show results for unsaturated lipids, in the absence of calcium (a and c) and in the presence of calcium (b and d). DPPE is represented by light gray triangles (▲), DSPE by medium gray squares (■), DOPE by dark gray diamonds (◆), and DDPE by black circles (●).

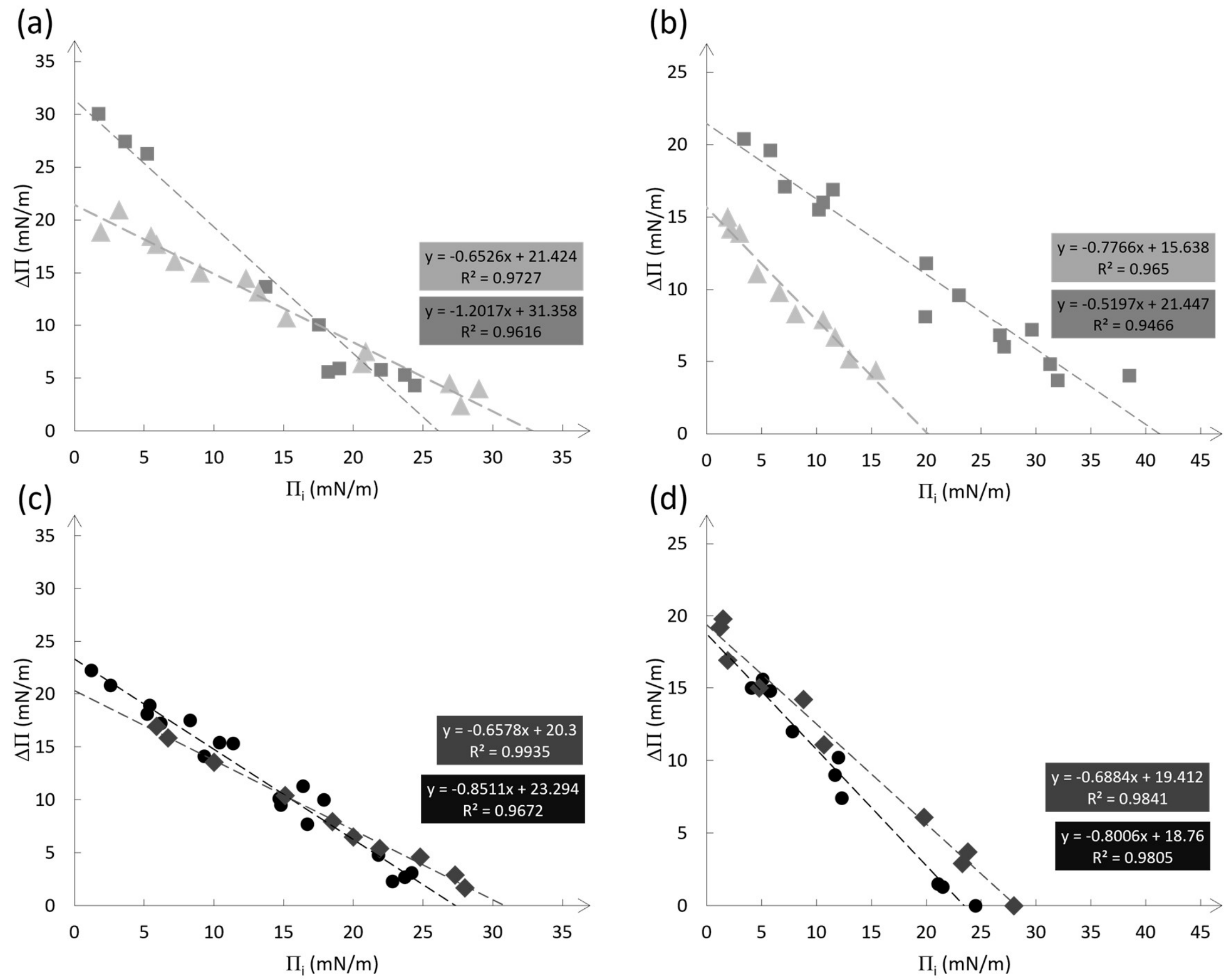

Figure S7. Linear regression of ΔΠ as a function of Π_i for determining the binding parameters of PC lipids. Panels (a) and (b) show results for saturated lipids, while panels (c) and (d) show results for unsaturated lipids, in the absence of calcium (a and c) and in the presence of calcium (b and d). DPPC is represented by light gray triangles (▲), DSPC by medium gray squares (■), DOPC by dark gray diamonds (◆), and DDPC by black circles (●).

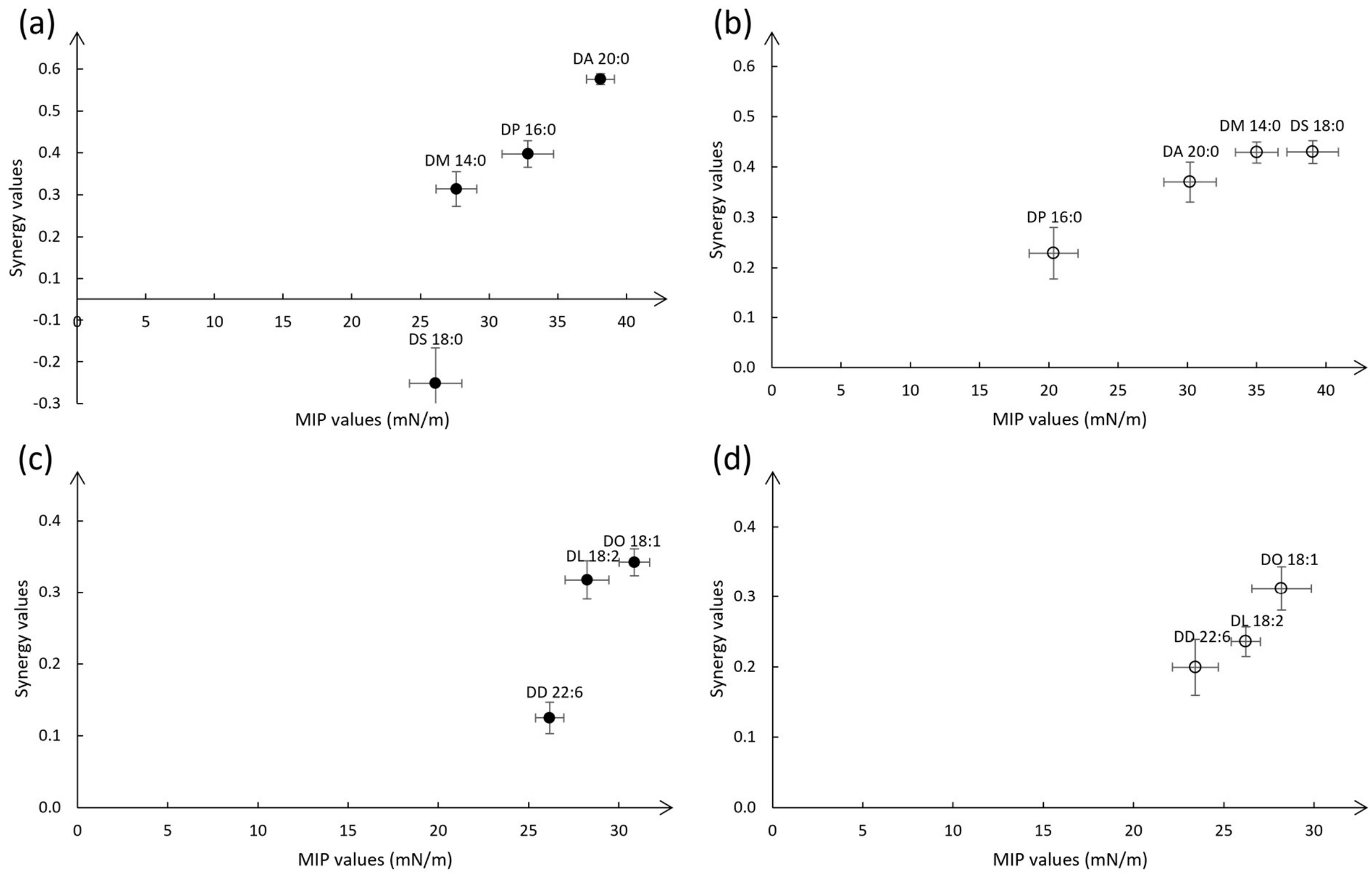

Figure S8. Correlation between synergy and MIP values for PC lipids: (a) saturated PC lipids without calcium, (b) saturated PC lipids with calcium, (c) unsaturated PC lipids without calcium, and (d) unsaturated PC lipids with calcium.

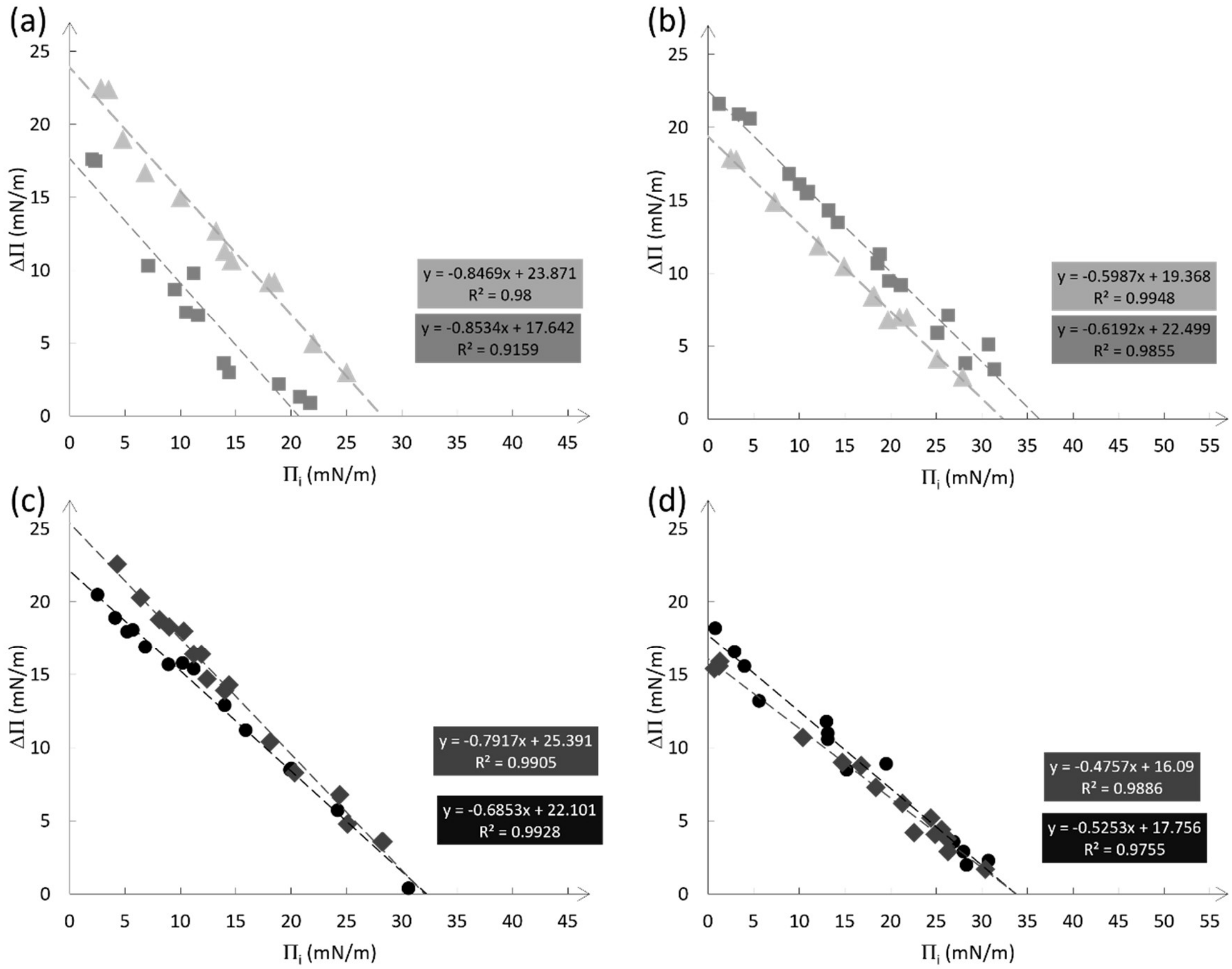

Figure S9. Linear regression of ΔΠ as a function of Π_i for determining the binding parameters of PS lipids. Panels (a) and (b) show results for saturated lipids, while panels (c) and (d) show results for unsaturated lipids, in the absence of calcium (a and c) and in the presence of calcium (b and d). DPPS is represented by light gray triangles (▲), DSPS by medium gray squares (■), DOPS by dark gray diamonds (◆), and DDPS by black circles (●).

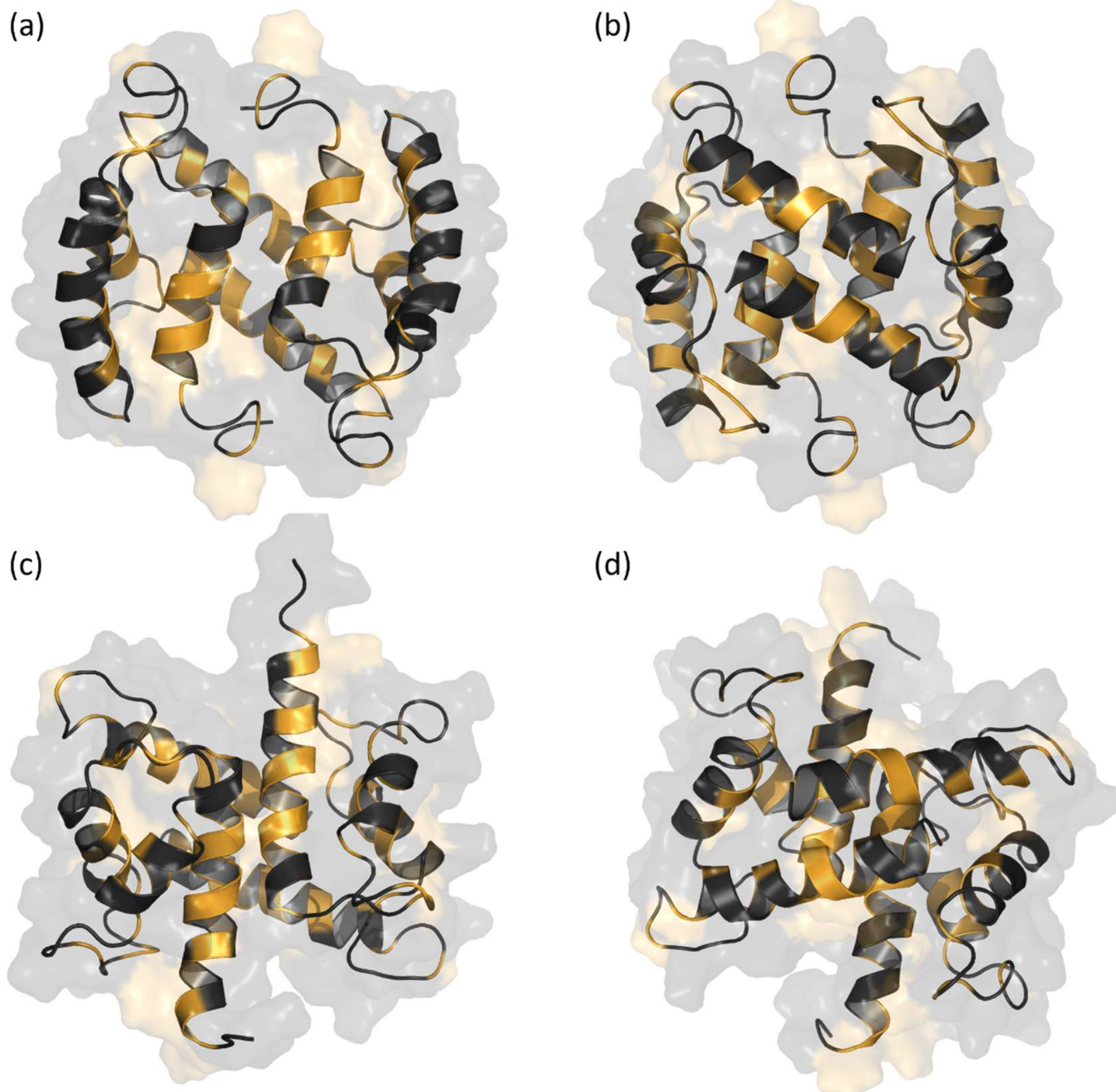

Figure S10. S100B PDB structures without (a, b) and with calcium ions (c, d). (a) apo S100B (PDB 2PRU) front and (b) back. (c) calcium-bound S100B (PDB 1UWO) front and (d) back. Yellow denotes residues with hydrophobic side chains.

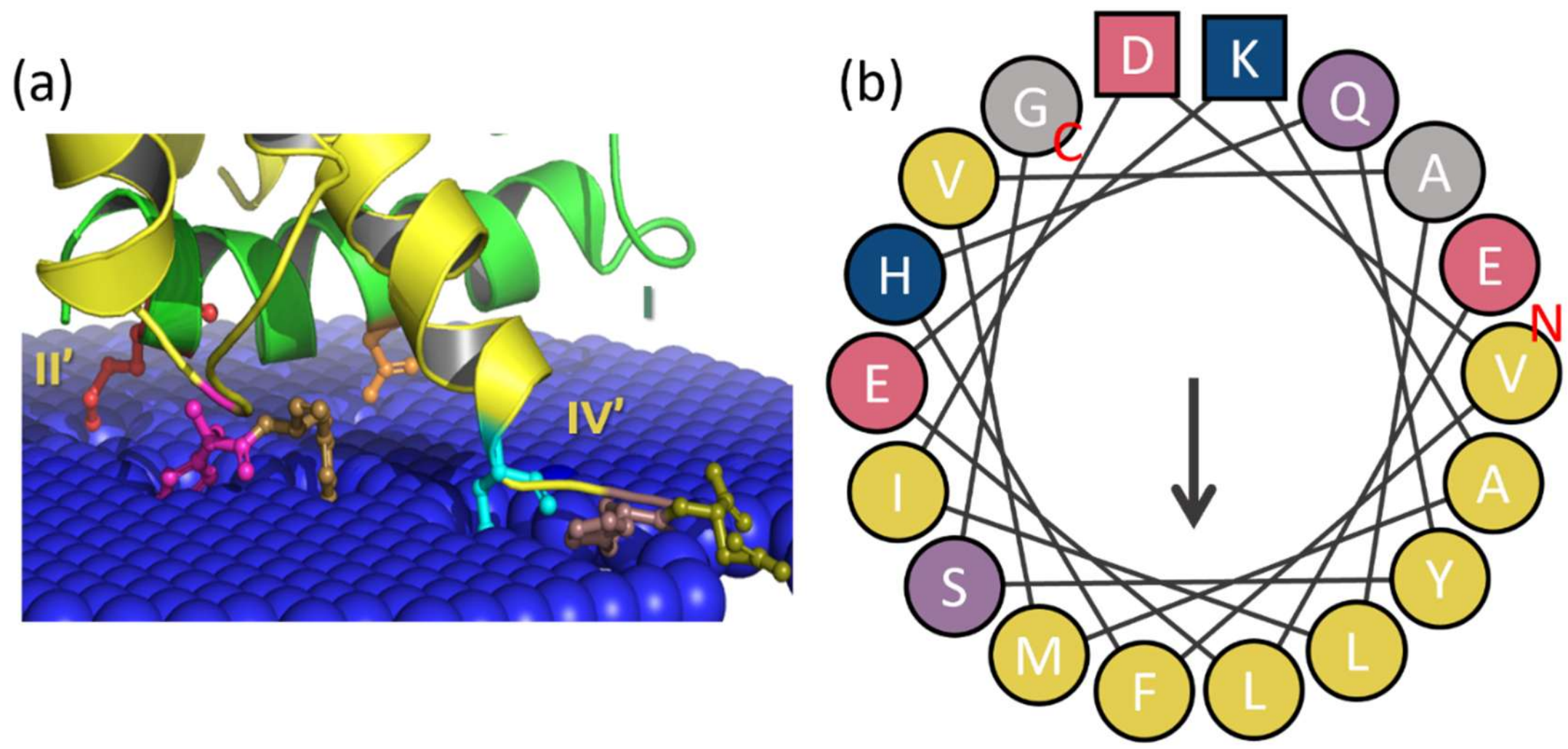

Figure S11. (a) Close-up simulation of the interaction between S100B and calcium ions with a model membrane generated by the PPM server. (b) Helicoidal plot of helix I of S100B, illustrating the hydrophobic face composed of the VAYLLFM residues predicted to participate in membrane interaction. Yellow denotes residues with hydrophobic side chains, grey denote glycine and alanine residues, blue denote positively charged residues, red denote negatively charged residues, and purple denote uncharged hydrophilic residues while normal residues are circle, lysine K5 and aspartic acid D12 predicted to be directly involved in membrane interaction are in square.

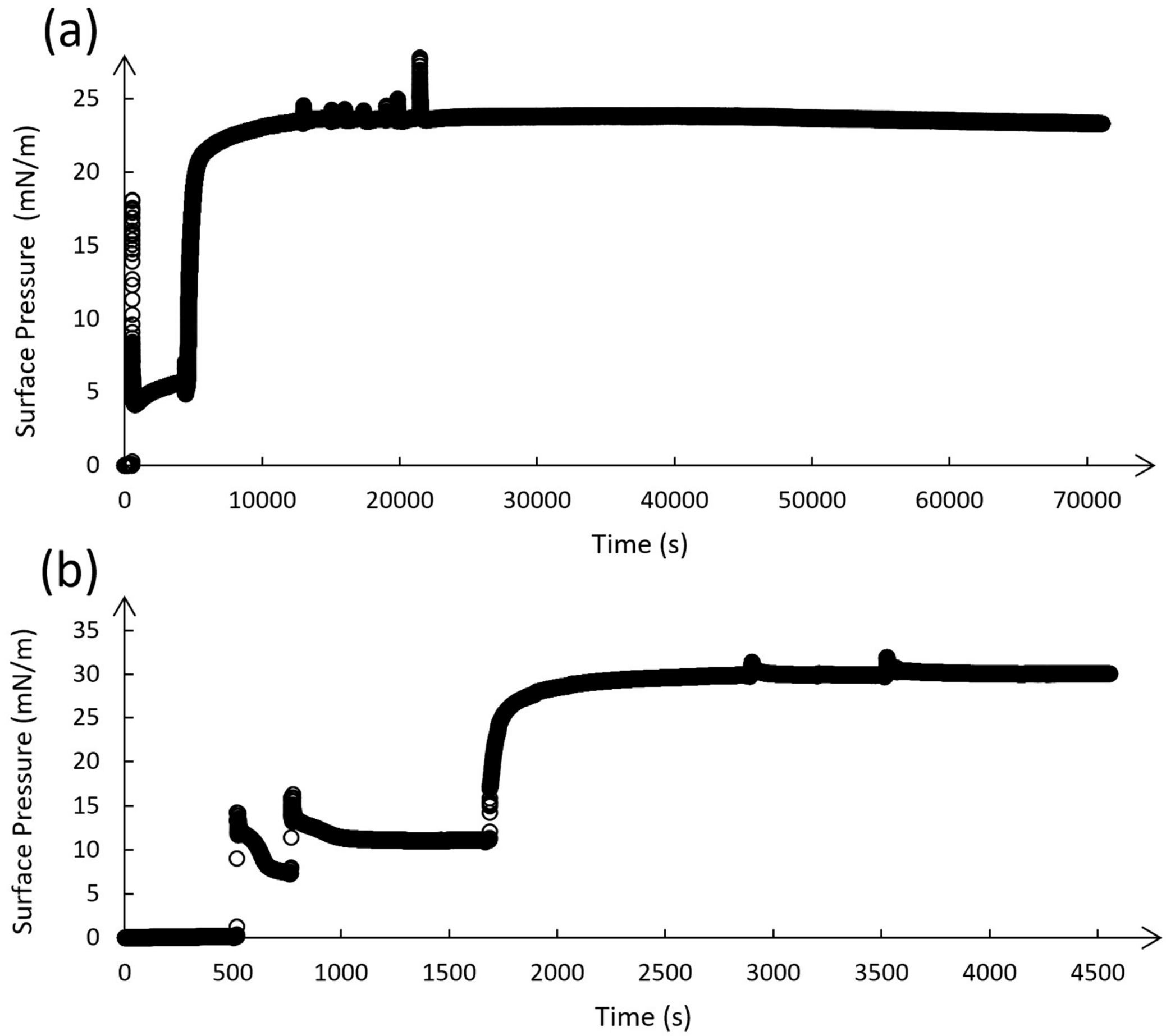

Figure S12. Surface pressure kinetics of S100B interactions with lipid monolayers. (a) Interaction with a DSPC monolayer in the absence of calcium ions. Surface pressure increased over time, reaching an equilibrium value of approximately 23.7 mN/m at around 13,000 seconds. Sequential injections of calcium ions were performed at approximately 13,050 seconds (1 mM final concentration), 15,500 seconds (2 mM), 16,600 seconds (3 mM), 18,250 seconds (4 mM), 19,060 seconds (5 mM), 19,860 seconds (10 mM), and 21,500 seconds (20 mM). Each injection caused a transient spike in surface pressure, which quickly returned to the equilibrium value of approximately 23.7 mN/m. (b) Interaction with a DSPS monolayer in the presence of calcium ions. Surface pressure increased over time, reaching an equilibrium value of approximately 29.8 mN/m at around 2,800 seconds.

Sequential injections of EDTA were performed at approximately 2,900 seconds (5 mM final concentration) and 3,550 seconds (15 mM). Each injection caused a transient spike in surface pressure, which quickly returned to near the equilibrium values of 29.8 mN/m and 30.0 mN/m, respectively. These observations suggest that once S100B is bound to the lipid monolayer, the interaction remains stable and is not readily reversible by the addition or removal of calcium ions under the conditions tested.

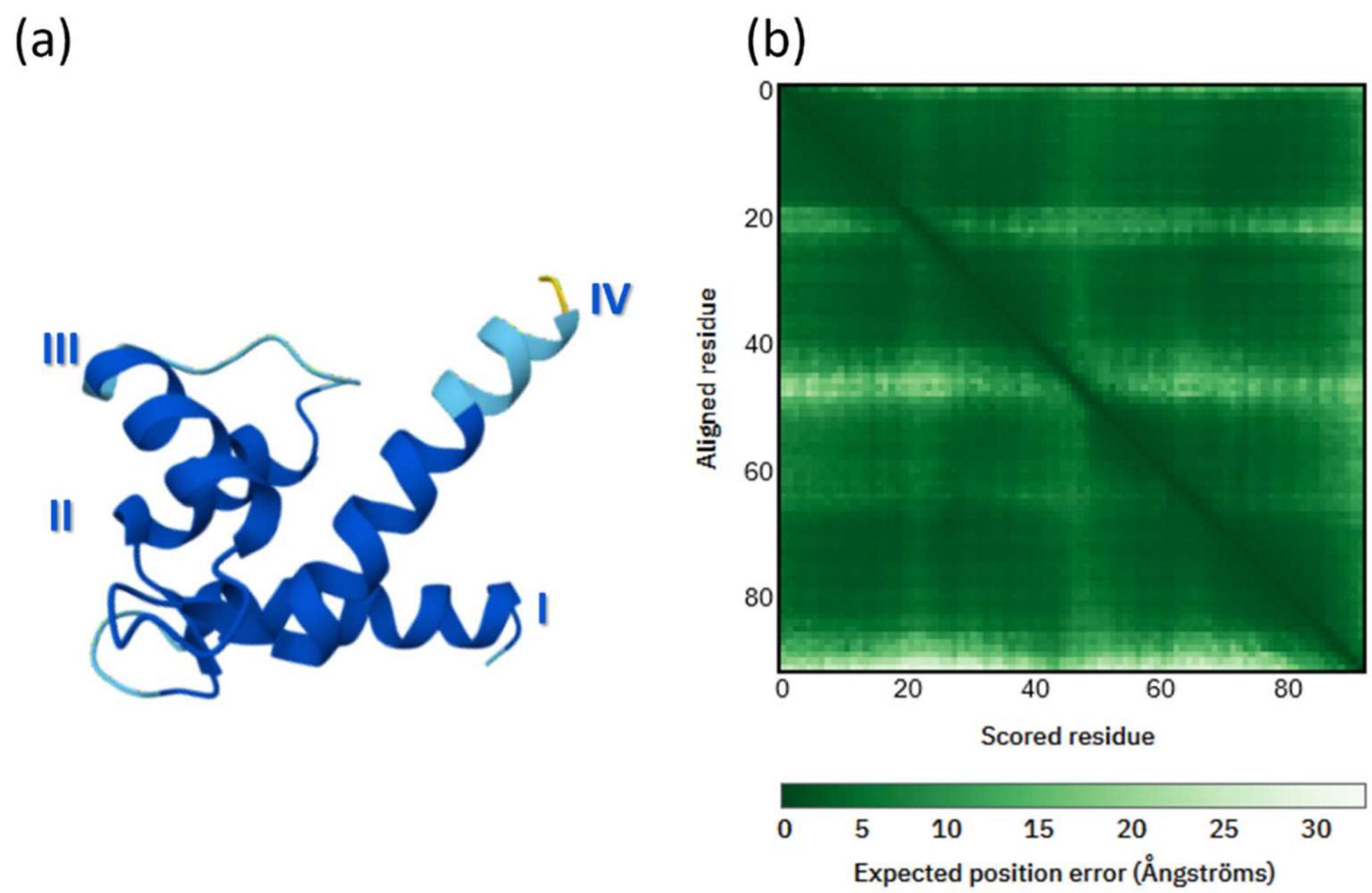

Figure S13: (a) Per-residue model confidence score (pLDDT) of monomeric S100B, dark blue residues have a very high pLDDT (over 90), light blue residues have a high pLDDT between 90 and 70, yellow residues have a low pLDDT (between 70 and 50) while orange residues (none here) have a very low pLDDT (below 50). (b) Predicted aligned error of monomeric S100B by AlphaFold which measures the confidence in the relative position of two residues within the predicted S100B structure with dark green tile corresponds to a good prediction (low error), whereas a light green tile indicates poor prediction (high error).

Table S1: MIP values (mN/m) of dimeric apo and calcium-bound S100B as well as monomeric apo-S100B.

MIP	PS	PE	PC
DP (16:0) – apo (dimeric S100B)	28.2 ± 1.5	32.7 ± 1.1	32.8 ± 1.9
DS (18:0) – apo (dimeric S100B)	20.7 ± 2.0	29.1 ± 1.6	26.1 ± 1.9
DO (18:1) – apo (dimeric S100B)	32.1 ± 0.9	29.4 ± 1.5	30.9 ± 0.8
DD (22:6) – apo (dimeric S100B)	32.3 ± 1.0	39.9 ± 2.2	26.2 ± 0.8
DP (16:0) - calcium-bound (dimeric S100B)	32.4 ± 0.8	37.4 ± 1.6	20.3 ± 1.8
DS (18:0) - calcium-bound (dimeric S100B)	36.9 ± 1.3	26.6 ± 1.9	39.0 ± 1.8
DO (18:1) - calcium-bound (dimeric S100B)	32.0 ± 2.0	38.8 ± 2.1	28.2 ± 1.6
DD (22:6) - calcium-bound (dimeric S100B)	33.8 ± 2.0	46.8 ± 1.8	23.4 ± 1.3
DD (22:6) – apo (monomeric S100B)	n.a.	34.3 ± 1.4	n.a.

Table S2: Synergy values of dimeric apo and calcium-bound S100B.

Synergy	PS	PE	PC
DP (16:0) - apo (dimeric S100B)	0.15 ± 0.04	0.21 ± 0.03	0.35 ± 0.03
DS (18:0) - apo (dimeric S100B)	0.15 ± 0.08	0.20 ± 0.04	-0.20 ± 0.08
DO (18:1) - apo (dimeric S100B)	0.21 ± 0.02	0.24 ± 0.03	0.34 ± 0.02
DD (22:6) - apo (dimeric S100B)	0.31 ± 0.02	0.52 ± 0.02	0.12 ± 0.02
DP (16:0) - calcium-bound (dimeric S100B)	0.40 ± 0.01	0.36 ± 0.03	0.23 ± 0.05
DS (18:0) - calcium-bound (dimeric S100B)	0.39 ± 0.02	0.19 ± 0.05	0.43 ± 0.02
DO (18:1) - calcium-bound (dimeric S100B)	0.50 ± 0.03	0.58 ± 0.02	0.31 ± 0.03
DD (22:6) - calcium-bound (dimeric S100B)	0.47 ± 0.03	0.59 ± 0.01	0.20 ± 0.04
DD (22:6) – apo (monomeric S100B)	n.a.	0.67 ± 0.01	n.a.
